# Nucleophilicities of Lewis Bases B and Electrophilicities of Lewis Acids A Determined from the Dissociation Energies of Complexes B⋯A Involving Hydrogen Bonds, Tetrel Bonds, Pnictogen Bonds, Chalcogen Bonds and Halogen Bonds

**DOI:** 10.3390/molecules22101786

**Published:** 2017-10-23

**Authors:** Ibon Alkorta, Anthony C. Legon

**Affiliations:** 1 Instituto de Química Médica (IQM-CSIC), Juan de la Cierva, 3, E-28006 Madrid, Spain; 2 School of Chemistry, University of Bristol, Cantock’s Close, Bristol BS8 1TS, UK

**Keywords:** noncovalent bonds, binary complexes, dissociation energies, nucleophilicity, electrophilicity, *ab initio* calculations, geometry

## Abstract

It is shown that the dissociation energy $D_{e}$ for the process B⋯A = B + A for 250 complexes B⋯A composed of 11 Lewis bases B (N_2_, CO, HC≡CH, CH_2_=CH_2_, C_3_H_6_, PH_3_, H_2_S, HCN, H_2_O, H_2_CO and NH_3_) and 23 Lewis acids (HF, HCl, HBr, HC≡CH, HCN, H_2_O, F_2_, Cl_2_, Br_2_, ClF, BrCl, H_3_SiF, H_3_GeF, F_2_CO, CO_2_, N_2_O, NO_2_F, PH_2_F, AsH_2_F, SO_2_, SeO_2_, SF_2_, and SeF_2_) can be represented to good approximation by means of the equation $D_{e}=c^{\prime }N_{B}E_{A}$, in which $N_{B}$ is a numerical nucleophilicity assigned to B, $E_{A}$ is a numerical electrophilicity assigned to A, and $c^{\prime }$ is a constant, conveniently chosen to have the value 1.00 kJ mol^−1^ here. The 250 complexes were chosen to cover a wide range of non-covalent interaction types, namely: (1) the hydrogen bond; (2) the halogen bond; (3) the tetrel bond; (4) the pnictogen bond; and (5) the chalcogen bond. Since there is no evidence that one group of non-covalent interaction was fitted any better than the others, it appears the equation is equally valid for all the interactions considered and that the values of $N_{B}$ and$\;E_{A}$ so determined define properties of the individual molecules. The values of $N_{B}$ and$\;E_{A}$ can be used to predict the dissociation energies of a wide range of binary complexes B⋯A with reasonable accuracy.

## 1. Introduction

The best known, earliest identified non-covalent interaction is the hydrogen bond, although the halogen bond was discovered (but not so called) as long ago as 1869 through the reaction of iodine with ammonia [1]. The pioneering experiments of Hassel and co-workers in the late 1950s and early 1960s [2] led to the recognition of the halogen bridge (the term used by Hassel for the halogen bond). This was followed by a lull in experimental work until the mid-1990s when there was a very rapid growth of interest [3,4] in what then became known as the halogen bond. In the last 10 years, several new types of non-covalent interaction have been named, including the tetrel bond [5], the pnictogen bond [6] and the chalcogen bond [7], although it has been pointed out [8,9] that these types of interaction were known for at least 30 years before they were assigned their current names.

Each non-covalent bond can be described [9,10] in terms of the interaction of an electrophilic region of one molecule with a nucleophilic region of another molecule (or even the same molecule). In this article, we shall concentrate on the pairwise interaction B⋯A of a Lewis base molecule B (the provider of the nucleophilic site, usually a non-bonding (n) or a π-bonding electron pair) and a Lewis acid A molecule (the provider of the electrophilic site). The electrophilic site in A can be identified variously as associated with a hydrogen atom (Group 1 of the Periodic Table), a tetrel atom (Group 14), a pnictogen atom (Group 15), a chalcogen atom (Group 16) or a halogen atom (Group 17); hence, the names assigned to the different types of non-covalent interaction. The electrophilic regions associated with halogen atoms in Lewis acids A were identified as positive regions on the molecular electrostatic surface potentials (MESP) by Clark, Murray and Politzer [11], who named the regions as σ-holes. Subsequently, σ-holes were similarly identified at or near Group 16 [12], 15 [13] and 14 [14] atoms in various Lewis acid molecules A.

An important aim in attempting to obtain a deeper understanding of non-covalent interactions in complexes B⋯A is to be able to predict the strength of the interaction in terms of the properties of the individual molecules B and A. Two measures of interaction strength have been recognized, namely the intermolecular stretching force constant $k_{\sigma }$ and the dissociation energy $D_{e}$ for the process B⋯A = B + A. The former is a measure of the energy $\frac{1}{2}k_{\sigma }$ required for a unit infinitesimal extension of the intermolecular bond from its equilibrium length, while the latter is the energy required for infinite extension of that bond. It was recognized some time ago [15] that experimental $k_{\sigma }$. values (determined, for example, by rotational spectroscopy [16] from centrifugal distortion constants) of weak hydrogen-bonded complexes B⋯HX (X = F, Cl, Br, CCH or CN) could be described by the following expression:

(1) $$k_{\sigma }=cN_{B}E_{A}$$

in which *c* = 2.5 N m^−1^ (a conveniently chosen constant) and $N_{B}$ and $E_{A}$ are the numerical nucleophilicity and electrophilicity of the Lewis base B and the Lewis acid A, respectively. It was shown later [3,17] that Equation 1 also applied to halogen-bonded complexes B⋯XY, where XY is a homo- or hetero-dihalogen molecule. A subsequent analysis [18] established that, for many hydrogen-bonded and halogen-bonded complexes B⋯HX and B⋯XY, the property $D_{e}$ (not generally available from experiment, but readily calculated *ab initio*) is directly proportional to the property $k_{\sigma }$ to a good level of accuracy and therefore that Equation 1 can be rewritten as

(2) $$D_{e}=c^{\prime }N_{B}E_{A}$$

where $c^{\prime }$ is another constant.

If the various types of non-covalent interaction referred to earlier can all be described through the interaction of a nucleophilic region of B with an electrophilic region of A, it should be possible to use Equation (2) to define a scale of nucleophilicities and electrophilicities for a wide range of Lewis bases B and Lewis acids A, whether held together in the complex B⋯A by a hydrogen bond, a tetrel bond, a pnictogen bond, a chalcogen bond or a halogen bond. In this article, we test this hypothesis for 11 Lewis bases and 23 Lewis acids. Thus, the values of $D_{e}$ for 250 complexes B⋯A covering the whole range of interaction types have been calculated *ab initio* at a high level of theory and fitted to Equation (2) (with the convenient choice $c^{\prime }=1.00$ kJ mol^−1^) to yield $N_{B}$ and $E_{A}$ values for the individual molecules. It is concluded that Equation (2) does indeed lead to a reliable scale of nucleophilicities and electrophilicities for the specified Lewis bases and acids.

## 2. Computational Methods

The geometries of the isolated monomers and complexes have been optimized at the MP2/aug-cc-pVTZ computational level [19,20,21] with the Gaussian-09 program [22]. Frequency calculations at the same level have been carried out to confirm that the resulting geometry corresponds to an energy minimum. Using these geometries, a series of calculations have been performed to derive the complete basis set (CBS) extrapolation at MP2 level. In addition, CCSD(T)/aug-cc-pVTZ calculations [23] have been carried out with the MOLPRO program [24] to derive the CCSD(T)/CBS energy. The extrapolation scheme is divided in the three parts. The first one corresponds to the Hartree–Fock (HF) contribution that is obtained using the aug-cc-pVTZ (AVTZ, X = 3) in Equations (3)–(6), aug-cc-pVQZ (AVQZ, X = 4) and aug-ccV5Z (AV5Z, X = 5) and Equations 1,2 [25]. In the second part, the MP2 correlation contribution is derived from the results obtained with MP2/aug-cc-pVTZ and MP2/aug-cc-pVQZ calculations and applying Equations (5)–(7) [26]. In the third part, higher order contributions are obtained as the difference of the CCSD(T)/aug-cc-pVTZ and MP2/aug-cc-pVTZ energies (Equation (6)) [27,28]. Finally, a CCSD(T)/CBS quality energy is generated by adding the $E_{\mathrm{CBS}}^{\mathrm{HF}}$, $E_{\mathrm{CBS}}^{\mathrm{MP}2\mathrm{corr}}$ and $\Delta E_{\mathrm{AVTZ}}^{\mathrm{CCSD}\left(T\right)}$ (Equation (9)). The latter energy has been obtained for all systems and used to calculate the dissociation energy, *D*_e_, of the complexes as the difference of the energy of the complex and the sum of the isolated monomers in their minimum geometry.

(3) $$E_{X}^{\mathrm{HF}}=E_{\mathrm{CBS}}^{\mathrm{HF}}+Ae^{-\mathrm{BX}}$$

(4) $$E_{\mathrm{CBS}}^{\mathrm{HF}}=E_{\mathrm{AVTZ}}^{\mathrm{HF}}-\frac{{\left(E_{\mathrm{AVTZ}}^{\mathrm{HF}}-E_{\mathrm{AVQZ}}^{\mathrm{HF}}\right)}^{2}}{\left(E_{\mathrm{AVTZ}}^{\mathrm{HF}}-2E_{\mathrm{AVQZ}}^{\mathrm{HF}}+E_{\mathrm{AV}5Z}^{\mathrm{HF}}\right)}$$

(5) $$E_{X}^{\mathrm{MP}2\mathrm{corr}}=E_{X}^{\mathrm{MP}2}-E_{X}^{\mathrm{HF}}$$

(6) $$E_{X}^{\mathrm{MP}2\mathrm{corr}}=E_{\mathrm{CBS}}^{\mathrm{MP}2\mathrm{corr}}+AX^{-3}$$

(7) $$E_{\mathrm{CBS}}^{\mathrm{MP}2\mathrm{corr}}=\frac{4^{3}E_{\mathrm{AVQZ}}^{\mathrm{MP}2\mathrm{corr}}-3^{3}E_{\mathrm{AVTZ}}^{\mathrm{MP}2\mathrm{corr}}}{4^{3}-3^{3}}$$

(8) $$\Delta E_{\mathrm{AVTZ}}^{\mathrm{CCSD}\left(T\right)}=E_{\mathrm{AVTZ}}^{\mathrm{CCSD}\left(T\right)}-E_{\mathrm{AVTZ}}^{\mathrm{MP}2}$$

(9) $$E_{\mathrm{CBS}}^{\mathrm{CCSD}\left(T\right)}=E_{\mathrm{CBS}}^{\mathrm{HF}}+E_{\mathrm{CBS}}^{\mathrm{MP}2\mathrm{corr}}+E_{\mathrm{AVTZ}}^{\mathrm{CCSD}\left(T\right)}$$

The *D*_e_ energies of all the complexes have been used to fit simultaneously the nucleophilicities and electrophilicities of the Lewis bases and Lewis acids studied by means of Equation (10).

(10) $$D_{e}=\left(\overset{\mathrm{Lewis}\;\mathrm{bases}}{\underset{i=1}{\sum }}x_{i}\times N_{Bi}\right)\times \left(\overset{\mathrm{Lewis}\;\mathrm{acids}}{\underset{j=1}{\sum }}x_{j}\times E_{Aj}\right)$$

The values of *x_i_* and *x_j_* are 1.0 when the corresponding Lewis base or Lewis acid is present in the complex, and 0.0 if it is absent.

The molecular electrostatic surface potentials (MESP) of the isolated Lewis bases and Lewis acids in their minimum-energy configurations have been calculated at the MP2/aug-cc-pVTZ computational level and analyzed with the Multiwfn [29,30] and DAMQT [31] programs. In the Lewis acids, the maximum value on the 0.001 au electron density isosurface associated with the interaction, *V*_S,max_, has been characterized. For the Lewis bases, two different values have been obtained. The first one, in analogy with the Lewis acids, is the minimum on the 0.001 au electron density isosurface, *V*_S,min_. The second value avoids the arbitrariness of the isosurface value by evaluation of the real minimum, *V*_min_.

## 3. Results

Table 1 displays values of $N_{B}$ for 11 Lewis bases and $E_{A}$ for 23 Lewis acids obtained from a least-squares fit of $D_{e}$ values calculated *ab initio* for 250 binary complexes B⋯A by using Equation (10). We actually carried out calculations of $D_{e}$ for the all of the 264 possible complexes that can be formed from the following 11 molecules B acting as Lewis bases: N_2_, CO, HC≡CH, CH_2_=CH_2_, C_3_H_6_ (cyclopropane), PH_3_, H_2_S, HCN, H_2_O, H_2_CO and NH_3_; and the following 24 molecules acting as Lewis acids: HF, HCl, HBr, HC≡CH, HCN, and H_2_O (constituting the hydrogen-bonded group); F_2_, Cl_2_, Br_2_, ClF, and BrCl (the halogen-bonded group); H_3_SiF, H_3_GeF, F_2_CO, and CO_2_ (the tetrel-bonded group); N_2_O, NO_2_F, PH_2_F, and AsH_2_F (the pnictogen-bonded group); and SO_2_, SO_3,_ SeO_2_, SF_2_, and SeF_2_ (the chalcogen-bonded group). The resulting $D_{e}$ values are given in Table 2, Table 3, Table 4, Table 5 and Table 6, respectively, for these various classes of non-covalent interaction. The reasons that 14 values were excluded from the global fit were as follows: (1) The complex H_3_P⋯ClF is known [32] to have significant ion-pair character [H_3_PCl]^+^⋯Cl^−^ with an enhanced $D_{e}\;$as a consequence and was excluded. (2) It was clear that several of the 11 complexes containing SO_3_ as a Lewis acid considerably worsened the fit. We noted that each B⋯SO_3_ complex had much a larger $D_{e}\;$value than any of its B⋯A counterparts, possibly a sign of significant ionic character and accordingly these too were excluded from the fit. (3) The complexes H_3_P⋯AsH_2_F and H_3_P⋯PH_2_F were significant outliers when included in the fit, perhaps because in these complexes each molecule can act simultaneously as a Lewis acid and a Lewis base [6,33], in contrast to the rest of the complexes where each molecule has only a single role. The values in Table 1 indicate that the order of nucleophilicities of the Lewis bases is:

N_2_ < CO < HC≡CH ~ PH_3_ < CH_2_=CH_2_ ~ C_3_H_6_ ~ H_2_S < HCN < H_2_O < H_2_CO < NH_3_
For the hydrogen-bonded complexes, the order of the electrophilicities of the Lewis acids is:
HF < HBr ~ HCl < HCN ~ H_2_O < HC≡CH
The order among the halogen-bonded systems is:
ClF > BrCl ~ Br_2_ > Cl_2_ > F_2_

The electrophilicity of the molecules forming chalcogen bonds via Se atoms are significantly larger than those involving S. Likewise, the AsH_2_F molecule has a larger electrophilicity and forms stronger pnictogen bonds than its P analogue, and the same order obtains for the propensity to form of tetrel bonds in H_3_GeF and H_3_SiF containing complexes.

**Figure 1:**
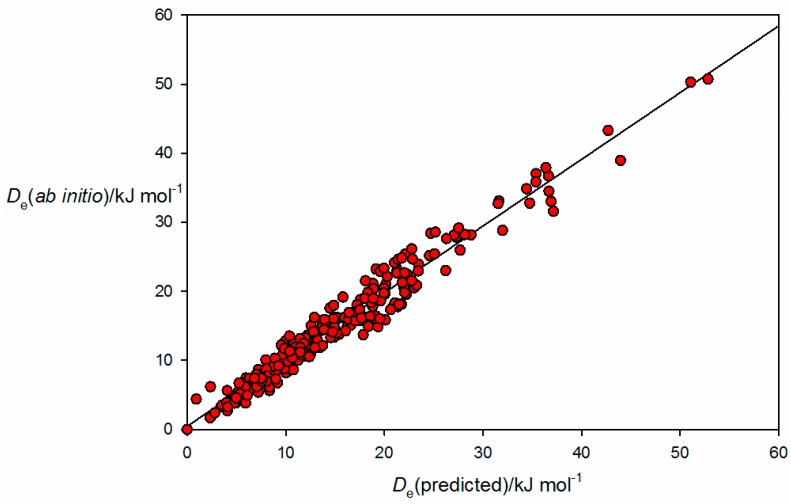
A graph of $D_{e}$ values of 250 complexes B⋯A calculated *ab initio* versus values of $D_{e}$ predicted from the nucleophilicities *N*_B_ and electrophilicites *E*_A_ of the 11 Lewis bases and the 24 Lewis acids, respectively (see Table 1), involved in the complexes.

A plot of $D_{e}$ from the *ab initio* calculations for the 250 complexes versus $D_{e}$ values generated from Equation (10) by using the $N_{B}$ and $E_{A}$ values given in Table 1 is shown in Figure 1. The correlation coefficient is 0.959 and indicates strongly that Equation (10) represents a good approximation for the 250 complexes included in the fit, a group that includes the sub-classes of complex in which the non-covalent interaction is a hydrogen bond, a halogen bond, a tetrel bond, a pnictogen bond and a chalcogen bond. Thus, it appears that Equation (2) (and therefore Equation (10)) provides a method of assigning nucleophilicities and electrophilicities to Lewis bases and Lewis acids, respectively, when involved in a wide range of non-covalent interactions. Conversely, the *N*_B_ and *E*_A_ values provide a method of predicting *D*_e_ for a given complex B⋯A.

**Figure 2:**
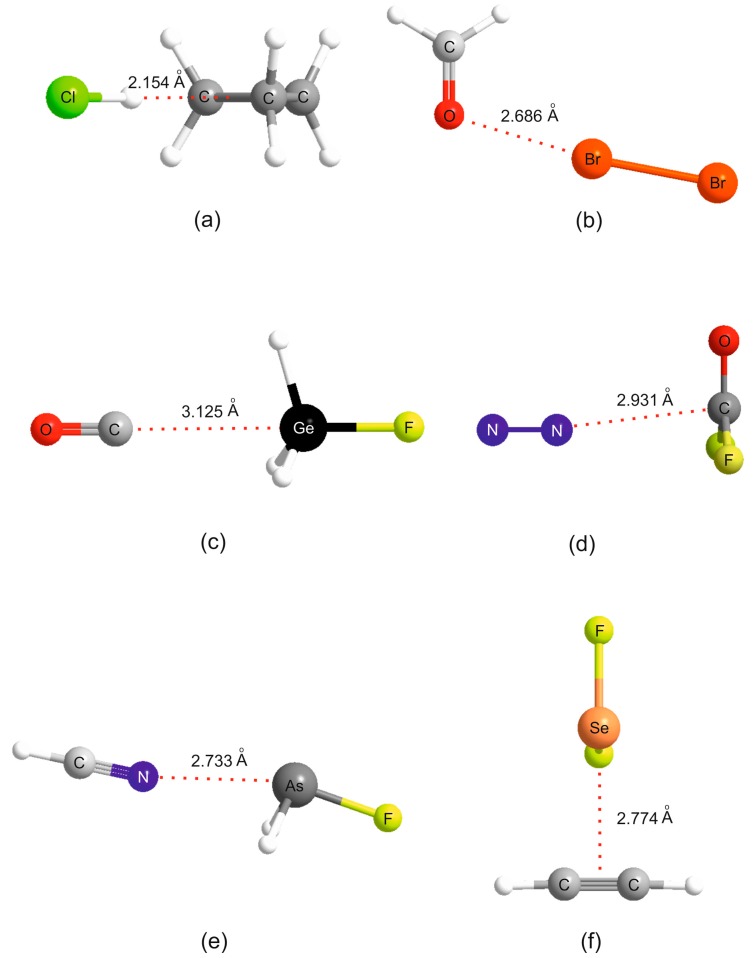
The geometries of selected complexes calculated *ab initio*. They provides examples of the following classes of non-covalent bonds: (**a**) a hydrogen-bond; (**b**) a halogen-bond; (**c**,**d**) different types of tetrel bond; (**e**) a pnictogen bond; and (**f**) a chalcogen bond. These examples are cited in the text when Figure 3, Figure 4, Figure 5, Figure 6, Figure 7 and Figure 8 are discussed.

**Figure 3:**
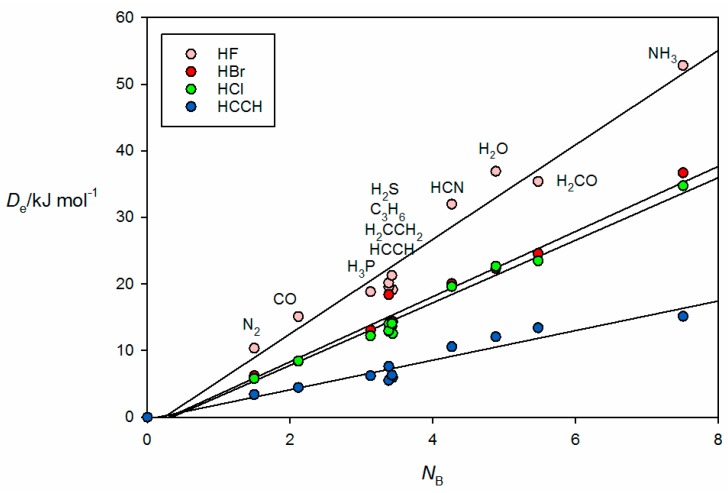
$D_{e}\;$ versus *N*_B_ for the series of hydrogen-bonded complexes B⋯HF,B⋯HCl, B⋯HBr and B⋯HC≡CH (see Table 1 for *N*_B_ of the indicated Lewis bases B).

**Figure 4:**
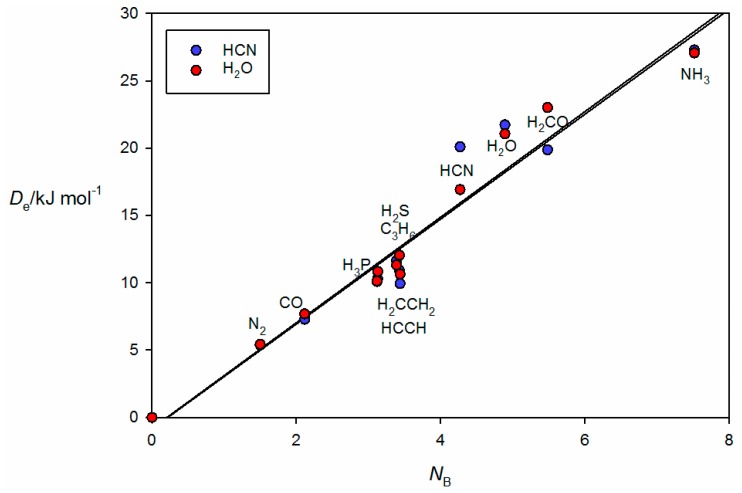
$D_{e}$ versus *N*_B_ for the series of hydrogen-bonded complexes B⋯HCN and B⋯HOH (see Table 1 for *N*_B_ of the indicated Lewis bases B).

It is instructive to discuss the behavior of the *ab initio* calculated $D_{e}$ values of the B⋯A within a given non-covalent interaction type with the *N*_B_ values determined by the least-squares fit (see Table 1). This discussion will refer to the *ab initio* geometries of the six selected complexes shown in Figure 2. Figure 3 shows the plot of $D_{e}$ versus *N*_A_ for 44 hydrogen bonded complexes B⋯HX formed from the 11 Lewis bases with HF, HCl, HBr and HC≡CH. The geometry determined for cyclopropane⋯HCl is shown in Figure 2a, where it is seen that the HCl molecules lies along a median of the cyclopropane equilateral triangle and therefore the electrophilic H atom of HCl samples one of the pseudo-π bonds of cyclopropane, in agreement with experiment [34]. Complexes involving HCN and H_2_O as the hydrogen donors are treated separately in Figure 4 because the points for the B⋯HCN and B⋯HOH complexes would be almost coincident with those associated with B⋯HCl and B⋯HBr if they were included in Figure 3. The individual straight lines represent the least-squares fit of the points for each B⋯HX series. The slope of each straight line is a measure of the electrophilicity of the given HX and corresponds approximately to the *E*_A_ value obtained in the global fit shown in Figure 1, as seen in Table 1.

We note that, in Figure 3 and Figure 4, there is a bunching of the points for B⋯HX, when B = PH_3_, H_2_S, HC≡CH, C_3_H_6_ and CH_2_=CH_2_ for each HX. The bunching is clearly systematic and independent of the HX molecule. Further, it is clear that the scatter of points from the appropriate straight line increases with the electrophilicity of HX.

**Figure 5:**
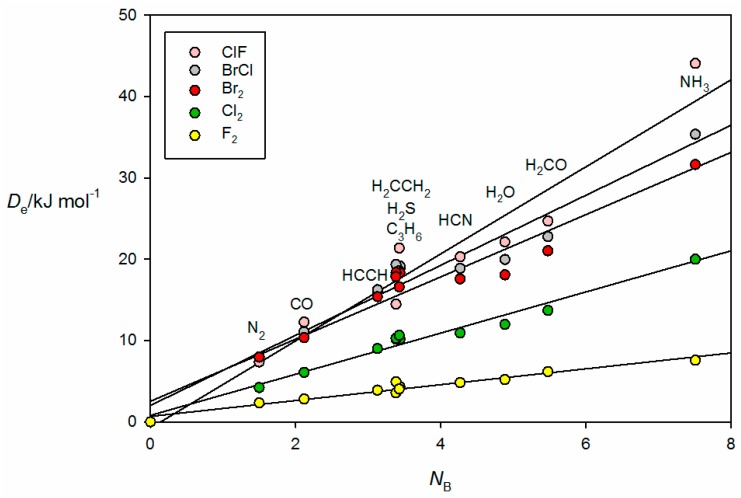
$D_{e}$ versus *N*_B_ for the series of halogen-bonded complexes B⋯XY, where XY = F_2_, Cl_2_, Br_2_, BrCl and ClF (see Table 1 for the *N*_B_ values of the indicated Lewis bases B).

The corresponding graph for the halogen-bonded complexes B⋯ClF, B⋯BrCl, B⋯Br_2_, B⋯Cl_2_ and B⋯F_2_ is displayed in Figure 5. Note that, for reasons already given, the result for H_3_P⋯ClF is omitted. The systematic bunching of complexes involving B = PH_3_, H_2_S, HC≡CH, C_3_H_6_ and CH_2_=CH_2_ for each XY and the increased scatter as the electrophilicity of the Lewis acid increases are again apparent for the B⋯XY. The geometry determined for H_2_CO⋯Br_2_ (see Figure 2b) suggests that the axial σ hole at each Br atom in the Br_2_ molecule lies along the direction of a nonbonding electron pair (n pair) on O, as conventionally envisaged. A similar geometry has been determined experimentally for H_2_CO⋯ClF [35]. Such interpretations led to sets of simple rules for predicting the geometries of hydrogen- and halogen-bonded complexes [3,36].

**Figure 6:**
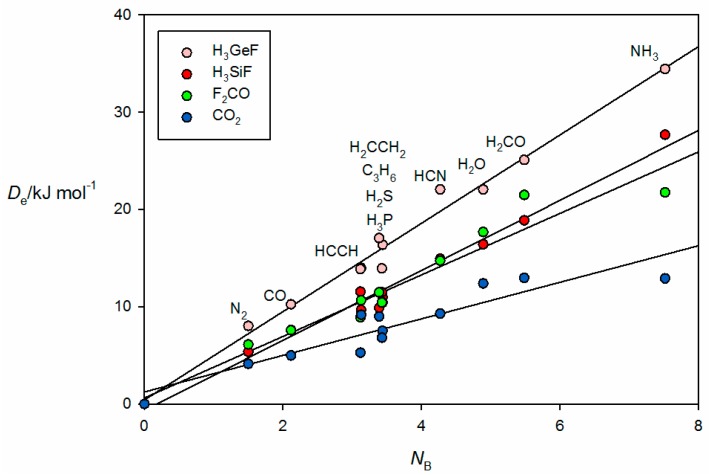
$D_{e}$ versus *N*_B_ for the series of tetrel-bonded complexes B⋯CO_2_, B⋯H_3_SiF, B⋯H_3_GeF and B⋯F_2_C=O (see Table 1 for the *N*_B_ values of the indicated Lewis bases B).

Figure 6 gives the results for tetrel-bonded complexes B⋯CO_2_, B⋯H_3_SiF, B⋯H_3_GeF and B⋯F_2_C=O when their *ab initio* values of $D_{e}$ are plotted against *N*_B_ of the 11 Lewis bases B. For the B⋯H_3_SiF and B⋯H_3_GeF complexes, the tetrel bond involves the n- or π-electron pairs of the Lewis base interacting with a σ hole that lies near the Si or Ge atom on the *C*_3_ axis, as can be clearly seen in the *ab initio* geometry determined for OC⋯H_3_GeF.shown in Figure 2c. The n-pair on the C atom of OC clearly interacts with the σ hole at Ge. F_2_CO forms tetrel bonds of a different type. This planar molecule has a π-hole at the carbon atom which is perpendicular to the molecular plane and the n- or π-pair of electrons of a Lewis base can interact with this, as is evident from the geometry determined for the N_2_⋯F_2_CO complex displayed in Figure 2d. A π-hole at C is also involved in the tetrel bond in B⋯CO_2_. The systematic features identified for hydrogen- and halogen-bonded complexes in Figure 3, Figure 4 and Figure 5 may also be seen in Figure 6.

**Figure 7:**
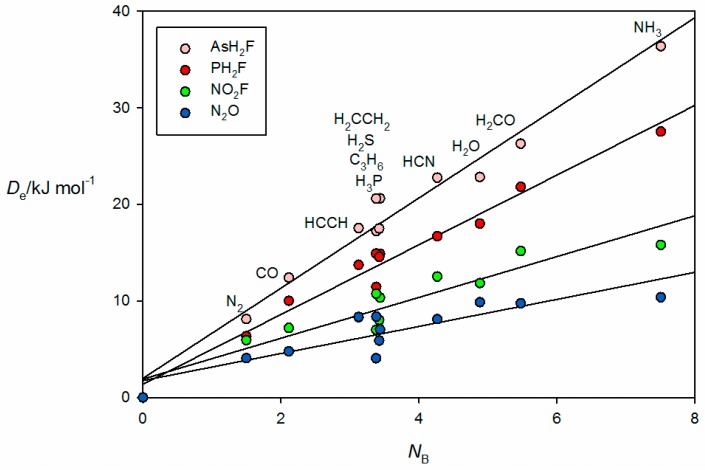
$D_{e}$ versus *N*_B_ for the series of pnictogen-bonded complexes B⋯PFH_2_, B⋯AsFH_2_, B⋯NO_2_F and B⋯N_2_O (see Table 1 for the *N*_B_ values of the indicated Lewis bases B).

The corresponding plots of $D_{e}$ against *N*_B_ for the pnictogen-bonded complexes B⋯PFH_2_, B⋯AsFH_2_, B⋯NO_2_F and B⋯N_2_O are given in Figure 7. The first two groups of pnictogen-bonded complexes utilize a σ hole near P or As along the P–F or As–F bond direction to form the non-covalent bond, while for the last two groups a π hole at the central N atom fulfills the role. The *ab initio* geometry of HCN⋯AsH_2_F in Figure 2e provides evidence of the σ-hole type of interaction. The pattern displayed by the *D*_e_ values of the pnictogen-bonded complexes in Figure 7 is similar to that seen in Figure 6 for the tetrel-bonded systems and again involves the characteristic bunching of points for the complexes in which B = PH_3_, H_2_S, HC≡CH, C_3_H_6_ and CH_2_=CH_2_. Recall that, for reasons given earlier, complexes H_3_P⋯PFH_2_ and H_3_P⋯AsFH_2_ were excluded from the global fit in Figure 1 and therefore from Figure 7.

**Figure 8:**
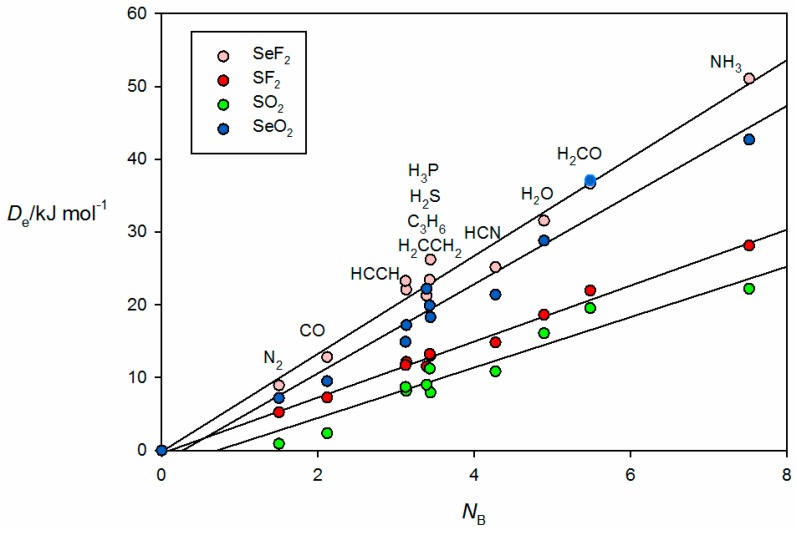
$D_{e}$ versus *N*_B_ for the series of chalcogen-bonded complexes B⋯SeF_2_, B⋯SF_2_, B⋯SO_2_ and B⋯SeO_2_ (see Table 1 for the *N*_B_ values of the indicated Lewis bases B).

The familiar bunching pattern is again observed in Figure 8, in which the *ab initio* calculated values of $D_{e}$ for complexes of the 11 Lewis bases B with each of the four Lewis acids SF_2_, SeF_2_, SO_2_ and SeO_2_ are plotted as a function of the *N*_B_ values of the various B. The complexes here all involve chalcogen bonds. In the B⋯SO_2_ and B⋯SeO_2_, the chalcogen bond is between a π hole at S or Se that is perpendicular to the plane containing the SO_2_ or SeO_2_ nuclei, but for B⋯SF_2_ and B⋯SeF_2_ a σ hole near S or Se at the termination of a S–F or Se–F bond is involved, as seen from the *ab initio* geometry of the complex formed by the interaction of π electrons of acetylene with the Se atom of SeF_2_ shown in Figure 2f.

It is of interest to ask whether there exists a linear relationship between the calculated *D*_e_values and the intermolecular distances in the various complexes B⋯A investigated. For this purpose, the intermolecular distances of the hydrogen-bonded complexes have been gathered in Table 7 while the intermolecular distances for the rest of the complexes are given in Tables S1–S4 (Supplementary Materials). (The full optimized geometries of all the systems are gathered in Table S5 of the Supplementary Materials). When seeking a linear relationship between the calculated *D*_e_ values (given in Table 2) and the intermolecular distances of all complexes a poor *R*^2^ value of 0.68 results. A more detailed analysis considering the individual hydrogen-bonded donors with all the bases (*n* = 11 for each correlation) reveals values of *R*^2^ between 0.80 and 0.53 (Table S6). Alternatively, looking to the correlation for a given base with the different HB donors (*n* = 5 for each correlation), leads to *R*^2^ values between 0.94 and 0.53. Similar results are obtained for the rest of the interactions considered here (Table S6). Thus, it is clear that the *D*_e_ values and the intermolecular distances are poorly correlated for the systems considered and consequently cannot be used to predict each other.

Parameters used in the literature to rationalize the *D*_e_ values are the molecular electrostatic potential values of the isolated bases (*V*_S,min_ and *V*_min_) or acids (*V*_S,max_) (see Section 2. for the definitions of the various quantities *V*). The corresponding values have been gathered in Table S7. The comparison of the *D_e_* values of the complexes of each Lewis acids with the *V*_S,min_ or *V*_min_ of the Lewis bases (*n* = 11 for each correlation) provide *R*^2^ values between 0.95 and 0.37 and between 0.97 and 0.43, respectively. Alternatively, the relationship of the V_S,max_ of the Lewis acids with the corresponding *D_e_* values for a single Lewis base (*n* = 24) show *R^2^* values between 0.75 and 0.25 (Table S8). Attempts to use simultaneously *V*_S,min_ of the Lewis bases and *V*_S,max_ of the Lewis acids in conjunction with the intermolecular distances for all the systems provide *R^2^* values smaller than 0.56. As in the case of the intermolecular distances, these parameters do not seem to be useful as predictive tool, save in closely related systems.

## 4. Conclusions

It has been shown that the dissociation energy $D_{e}$ for the reaction B⋯A = B + A for 250 complexes B⋯A composed of 11 Lewis bases B and 23 Lewis acids can be represented to good approximation by means of the equation $D_{e}=c^{\prime }N_{B}E_{A}$, in which $N_{B}$ is a numerical nucleophilicity assigned to B, $E_{A}$ is a numerical electrophilicity assigned to A, and $c^{\prime }$ is a constant, conveniently chosen to have the value 1.00 kJ mol^−1^ here. The 250 complexes were chosen to cover a wide range of non-covalent interaction types, namely: (1) the hydrogen bond; (2) the halogen bond; (3) the tetrel bond; (4) the pnictogen bond; and (5) the chalcogen bond. There was no evidence that one group of non-covalent interaction was fitted any better than the others. Therefore, the equation appears to be equally valid for all the interactions considered here and the values of $N_{B}$ and$\;E_{A}$ determined in the manner discussed appear to be valid properties of the individual molecules. The values of $N_{B}$ and$\;E_{A}$ can therefore be used from to predict the dissociation energies of a wide range of binary complexes B⋯A with reasonable accuracy.

If the weak interaction in the complexes B⋯A considered here is predominantly electrostatic in nature, it seems reasonable that the individual molecular properties $N_{B}$ and$\;E_{A}$ are related to the molecular electrostatic surface potentials in the regions of the molecules to which these properties apply. We attempted to test this by examining the product of the maximum positive surface potential (to represent the electrophilic region of molecule A) and the maximum negative surface potential (to represent the nucleophilic region of molecule B) but the correlation was poor.

## Figures and Tables

**Table 1:** Values of nucleophilicities *N*_B_ of 11 Lewis bases B and electrophilicities *E*_A_ of 23 Lewis acids A obtained by fitting the dissociation energies $D_{e}$ of 250 complexes B⋯A to Equation (10).

Lewis Base		Lewis Acid		Lewis Acid	
B	*N*_B_	A	*E*_A_	A	*E*_A_
N_2_	1.50	HF	6.75	SeO_2_	5.76
C≡O	2.12	HBr	4.59	SeF_2_	6.69
HC≡CH	3.13	HCl	4.36	SF_2_	3.75
H_2_C=CH_2_	3.44	HC≡N	3.71	SO_2_	2.92
C_3_H_6_	3.39	H_2_O	3.74	AsH_2_F	5.04
PH_3_	3.12	HC≡CH	2.16	PH_2_F	3.88
H_2_S	3.43	ClF	5.18	NO_2_F	2.55
HC≡N	4.27	BrCl	4.77	N_2_O	1.80
H_2_C= O	5.48	Br_2_	4.40	GeH_3_F	4.63
H_2_O	4.89	Cl_2_	2.71	SiH_3_F	3.45
NH_3_	7.52	F_2_	1.13	F_2_C=O	3.30
				CO_2_	2.15

**Table 2:** Values of *D*_e_/(kJ mol^−1^) for 66 hydrogen-bonded complexes calculated *ab initio*.

Lewis Base	Lewis Acid					
	HF	HBr	HCl	HC≡N	H_2_O	HC≡CH
N_2_	10.34	6.21	5.81	5.37	5.43	3.40
CO	15.09	8.39	8.44	7.28	7.68	4.45
HC≡CH	18.84	13.07	12.22	10.30	10.83	6.22
H_2_C=CH_2_	19.15	14.34	12.56	9.93	10.64	5.96
C_3_H_6_	19.57	18.39	14.00	11.63	11.31	7.62
PH_3_	20.15	12.90	12.97	10.10	10.13	5.51
H_2_S	21.29	13.63	14.06	10.92	12.03	6.30
HC≡N	31.99	20.06	19.60	20.09	16.92	10.56
H_2_C=O	35.38	24.57	23.46	19.87	23.01	13.42
H_2_O	36.91	22.33	22.65	21.72	21.06	12.05
NH_3_	52.81	36.69	34.75	27.28	27.07	15.15

**Table 3:** Values of *D*_e_/(kJ mol^−1^) for 55 halogen-bonded complexes calculated *ab initio*.

Lewis Base	Lewis Acid				
	ClF	BrCl	Br_2_	Cl_2_	F_2_
N_2_	7.35	7.98	7.93	4.20	2.33
CO	12.25	11.06	10.35	6.08	2.80
HC≡CH	15.96	16.23	15.38	8.99	3.89
H_2_C=CH_2_	18.79	19.14	18.33	10.16	4.31
C_3_H_6_	14.46	18.16	18.36	10.18	4.92
PH_3_	38.56	19.35	17.85	10.28	3.54
H_2_S	21.36	18.56	16.58	10.63	4.02
HC≡N	20.28	18.87	17.56	10.93	4.82
H_2_C=O	24.70	22.76	21.02	13.69	6.16
H_2_O	22.11	19.95	18.06	11.97	5.20
NH_3_	43.96	35.37	31.63	19.98	7.57

**Table 4:** Values of *D*_e_/(kJ mol^−1^) for 44 tetrel-bonded complexes calculated *ab initio*.

Lewis Base	Lewis Acid			
	GeH_3_F	SiH_3_F	F_2_C=O	CO_2_
N_2_	8.02	5.36	6.11	4.11
CO	10.23	7.59	7.57	4.97
HC≡CH	13.97	9.66	10.65	9.16
H_2_C=CH_2_	16.36	10.96	10.41	7.51
C_3_H_6_	17.05	9.88	11.47	8.99
PH_3_	13.86	11.53	8.90	5.26
H_2_S	13.93	11.48	10.41	6.80
HC≡N	22.05	14.92	14.72	9.27
H_2_C=O	25.09	18.87	21.49	12.96
H_2_O	22.04	16.41	17.67	12.38
NH_3_	34.42	27.67	21.74	12.90

**Table 5:** Values of *D*_e_/(kJ mol^−1^) for 44 pnictogen-bonded complexes calculated *ab initio*.

Lewis Base	Lewis Acid			
	AsH_2_F	PH_2_F	NO_2_F	N_2_O
N_2_	8.11	6.33	5.93	4.06
CO	12.41	10.00	7.19	4.77
HC≡CH	17.54	13.73	8.31	8.33
H_2_C=CH_2_	20.62	14.88	10.33	7.04
C_3_H_6_	17.23	11.44	10.75	8.35
PH_3_	27.79	20.90	7.00	4.05
H_2_S	17.50	14.55	8.02	5.88
HC≡N	22.76	16.69	12.51	8.11
H_2_C=O	26.28	21.81	15.15	9.76
H_2_O	22.84	18.02	11.83	9.86
NH_3_	36.37	27.53	15.79	10.37

**Table 6:** Values of *D*_e_/(kJ mol^−1^) for 55 chalcogen-bonded complexes calculated *ab initio*.

Lewis Base	Lewis Acid				
	SO_3_	SeF_2_	SeO_2_	SF_2_	SO_2_
N_2_	6.30	8.95	7.21	5.24	0.92
CO	12.56	12.81	9.53	7.26	2.36
HC≡CH	19.92	22.08	17.21	12.14	8.17
H_2_C=CH_2_	23.30	26.21	18.31	13.03	7.97
C_3_H_6_	17.11	21.27	22.21	11.54	9.03
PH_3_	43.73	23.27	14.86	11.72	8.71
H_2_S	31.71	23.42	19.94	13.23	11.23
HC≡N	28.48	25.16	21.40	14.82	10.86
H_2_C=O	43.80	36.64	37.14	21.95	19.53
H_2_O	37.89	31.55	28.78	18.63	16.07
NH_3_	99.23	51.06	42.70	28.14	22.20

**Table 7:** Intermolecular distances *r*(B⋯H)/Å between the atom of the B and the H atom of A involved in the hydrogen-bond interaction in 66 hydrogen-bonded complexes.

Lewis Base	Lewis Acid					
	HF	HBr	HCl	HC≡N	H_2_O	HC≡CH
N_2_	2.055	2.342	2.297	2.412	2.318	2.499
C≡O	2.057	2.355	2.301	2.490	2.337	2.600
HC≡CH	2.125	2.301	2.292	2.498	2.348	2.602
H_2_C=CH_2_	2.129	2.309	2.297	2.540	2.367	2.645
C_3_H_6_	2.024	2.158	2.154	2.314	2.421	2.249
PH_3_	2.336	2.519	2.505	2.794	2.615	2.926
H_2_S	2.268	2.434	2.416	2.670	2.513	2.795
HC≡N	1.835	2.052	2.015	2.186	2.089	2.321
H_2_C=O	1.708	1.840	1.830	2.075	1.972	2.225
H_2_O	1.704	1.893	1.863	2.044	1.945	2.188
NH_3_	1.679	1.687	1.738	2.102	1.957	2.259

## Supplementary Materials

The supplementary materials are available online. Table S1: Intermolecular distances *r*(B⋯X)/Å between the atom of the B and the X atom of A involved in the Halogen-bond interaction in 55 halogen-bonded complexes, Table S2: Intermolecular distances *r*(B⋯X)/Å between the atom of the B and the T atom of A involved in the tetrel-bond interaction in 44 tetrel-bonded complexes, Table S3: Intermolecular distances *r*(B⋯X)/Å between the atom of the B and the Z atom of A involved in the pnictogen-bond interaction in 44 pnictogen-bonded complexes, Table S4: Intermolecular distances *r*(B⋯X)/Å between the atom of the B and the Y atom of A involved in the chalcogen-bond interaction in 55 chalcogen-bonded complexes, Table S5: Optimized geometries (Å, °) and energies (Hartree) at MP2/aug-cc-pVTZ computational level, Table S6: Linear correlations of *D*_e_ vs. the interatomic distance (R^2^ coefficients), Table S7: V_S,min_ and V_min_ of the Lewis Bases and V_S,max_ of the Lewis acids. The 0.001 au electron density isosurface has been chosen to calculate V_S,min_ and V_S,max_, Table S8: Linear correlations of *D*_e_ vs. the MEP parameters (V_S,max_, V_S,min_ and V_min_)(R^2^ coefficients).
