# Supplementary material for: Nucleophilicities of Lewis Bases B and Electrophilicities of Lewis Acids A Determined from the Dissociation Energies of Complexes B⋯A Involving Hydrogen Bonds, Tetrel Bonds, Pnictogen Bonds, Chalcogen Bonds and Halogen Bonds
Source: Molecules. 2017 Oct 23;22(10):1786. doi: 10.3390/molecules22101786 (PMC6151704; doi:10.3390/molecules22101786)
Supplement: Supplementary file 1 [file molecules-22-01786-s001.pdf]

Supporting Information

Nucleophilicities of Lewis bases B and electrophilicities of Lewis acids A determined from the dissociation energies of weakly bound complexes B···A

Ibon Alkorta and Anthony C. Legon

Pg. S2	Table S1. Intermolecular distances *r*(B···X)/Å between the atom of the B and the X atom of A involved in the Halogen-bond interaction in 55 halogen-bonded complexes.
Pg. S2	Table S2. Intermolecular distances *r*(B···T)/Å between the atom of the B and the T atom of A involved in the tetrel-bond interaction in 44 tetrel-bonded complexes.
Pg. S3	Table S3. Intermolecular distances *r*(B···Z)/Å between the atom of the B and the Z atom of A involved in the pnictogen-bond interaction in 44 pnictogen-bonded complexes.
Pg. S3	Table S4. Intermolecular distances *r*(B···Y)/Å between the atom of the B and the Y atom of A involved in the chalcogen-bond interaction in 55 chalcogen-bonded complexes.
Pg. S4-S73	Table S5. Optimized geometries (Å, °) and energies (Hartree) at MP2/aug-cc-pVTZ computational level.
Pg.S74-75	Table S6. Linear correlations of D_e vs. the interatomic distance (R^2 coefficients)
Pg. S76	Table S7. $V_{S,min}$ and V_{min} of the Lewis Bases and $V_{S,max}$ of the Lewis acids. The 0.001 au electron density isosurface has been chosen to calculate $V_{S,min}$ and $V_{S,max}$.
Pg. S77	Table S8. Linear correlations of D_e vs. the MEP parameters ($V_{S,max}$, $V_{S,min}$ and V_{min}) (R^2 coefficients)

Table S1. Intermolecular distances *r*(B···X)/Å between the atom of the B and the X atom of A involved in the Halogen-bond interaction in 55 halogen-bonded complexes.

Lewis base	Lewis acid				
	ClF	ClBr	Br_2	Cl_2	F_2
N_2	2.803	2.979	3.038	3.018	2.823
CO	2.661	2.899	2.987	3.036	2.879
HC≡CH	2.728	2.907	2.965	3.000	2.855
$H_2C=CH_2$	2.510	2.742	2.815	2.892	2.768
C_3H_6	2.824	2.946	2.978	2.980	2.800
H_3P	2.183	2.659	2.836	3.050	3.048
H_2S	2.721	2.972	3.045	3.100	3.021
HN≡C	2.541	2.722	2.789	2.822	2.706
$H_2C=O$	2.432	2.627	2.686	2.700	2.604
H_2O	2.517	2.698	2.757	2.774	2.649
H_3N	2.233	2.469	2.538	2.592	2.594

Table S2. Intermolecular distances *r*(B···T)/Å between the atom of the B and the T atom of A involved in the tetrel-bond interaction in 44 tetrel-bonded complexes.

Lewis base	Lewis acid			
	GeH_3F	SiH_3F	$F_2C=O$	CO_2
N_2	3.089	3.175	2.931	3.089
CO	3.125	3.169	3.027	3.180
HC≡CH	3.257	3.308	3.109	3.161
$H_2C=CH_2$	3.178	3.225	3.116	3.236
C_3H_6	3.228	3.343	3.058	3.101
H_3P	3.402	3.406	3.352	3.468
H_2S	3.370	3.380	3.272	3.398
HN≡C	2.844	2.849	2.769	2.946
$H_2C=O$	2.699	2.660	2.592	2.837
H_2O	2.777	2.766	2.650	2.773
H_3N	2.641	2.498	2.676	2.937

Table S3. Intermolecular distances r(B···Z)/Å between the atom of the B and the Z atom of A involved in the pnictogen-bond interaction in 44 pnictogen-bonded complexes.

	Lewis acid			
Lewis base	AsH_2F	PH_2F	NO_2F	N_2O
N_2	2.954	2.999	2.907	3.035
CO	2.899	2.910	3.008	3.138
HC≡CH	3.107	3.150	3.144	3.112
$H_2C=CH_2$	3.007	3.035	3.075	3.169
C_3H_6	3.108	3.183	3.070	3.086
H_3P	3.082	3.060	3.310	3.450
H_2S	3.163	3.196	3.306	3.397
HN≡C	2.733	2.775	2.817	2.953
$H_2C=O$	2.636	2.645	2.711	2.891
H_2O	2.714	2.750	2.741	2.825
H_3N	2.596	2.608	2.822	3.029

Table S4. Intermolecular distances r(B···Y)/Å between the atom of the B and the Y atom of A involved in the chalcogen-bond interaction in 55 chalcogen-bonded complexes.

Lewis base	Lewis acid				
	SO_3	SeF_2	SeO_2	SF_2	SO_2
N_2	2.864	2.948	3.280	3.057	3.290
CO	2.808	2.877	3.333	3.059	3.367
HC≡CH	2.887	2.774	3.238	3.023	3.268
$H_2C=CH_2$	2.809	2.566	3.273	2.906	3.331
C_3H_6	2.897	2.960	3.040	3.059	3.100
H_3P	2.501	2.904	3.347	3.197	3.513
H_2S	2.784	2.994	3.311	3.159	3.415
HN≡C	2.547	2.650	2.968	2.799	3.013
$H_2C=O$	2.275	2.507	2.671	2.621	2.742
H_2O	2.375	2.550	2.764	2.669	2.850
H_3N	2.017	2.388	2.617	2.482	2.763

Table S5. Optimized geometries (Å, °) and energies (Hartree) at MP2/aug-cc-pVTZ computational level.

Molecules acting as Lewis Bases (LB)

n2 MP2= -109.36479979 NIMAG= 0
N
N,1,r1

r1=1.11404452

co MP2= -113.14241107 NIMAG= 0
C
O,1,r1

r1=1.13895962

hcch MP2= -77.16405740 NIMAG= 0
X
X,1,1.
C,1,r1,2,90.
C,1,r1,2,90.,3,180.,0
H,1,r2,2,90.,3,0.,0
H,1,r2,2,90.,3,180.,0

r1=0.60607914
r2=1.66782079

h2cch2 MP2= -78.40452910 NIMAG= 0
X
X,1,1.
C,1,r1,2,90.
C,1,r1,2,90.,3,180.,0
H,3,r2,1,a2,2,0.,0
H,3,r2,1,a2,2,180.,0
H,4,r2,1,a2,2,0.,0
H,4,r2,1,a2,2,180.,0

r1=0.66659638
r2=1.08097374
a2=121.32754008

c3h6 MP2= -117.62547653 NIMAG= 0
X
C,1,r1
C,1,r1,2,120.
C,1,r1,2,120.,3,180.,0
H,2,r2,1,a2,3,90.,0
H,2,r2,1,a2,3,-90.,0
H,3,r2,1,a2,2,90.,0
H,3,r2,1,a2,2,-90.,0

H,4,r2,1,a2,3,90.,0
H,4,r2,1,a2,3,-90.,0

r1=0.86891209
r2=1.07896943
a2=122.44404144

ph3 MP2= -342.66128825 NIMAG= 0
P
X,1,1.
H,1,r1,2,a1
H,1,r1,2,a1,3,120.,0
H,1,r1,2,a1,3,-120.,0

r1=1.41240297
a1=122.65471825

sh2 MP2= -398.90881780 NIMAG= 0
S
H,1,roh
H,1,roh,2,ahoh

roh=1.3360337
ahoh=92.21579148

nch MP2= -93.25974985 NIMAG= 0
C
X,1,1.
N,1,r1,2,90.
H,1,r2,2,90.,3,180.,0

r1=1.16695694
r2=1.06463634

ch2o MP2= -114.31640998 NIMAG= 0
C
O,1,r1
H,1,r2,2,a2
H,1,r2,2,a2,3,180.,0

r1=1.21312079
r2=1.10005812
a2=121.67768332

oh2 MP2= -76.32899232 NIMAG= 0
O
H,1,roh
H,1,roh,2,ahoh

roh=0.96133268
ahoh=104.10907946

nh3 MP2= -56.46054087 NIMAG= 0
N
X,1,1.
H,1,r1,2,a1
H,1,r1,2,a1,3,120.,0
H,1,r1,2,a1,3,-120.,0

r1=1.01212363
a1=112.05948587

HB acids
fh MP2= -100.34089069 NIMAG= 0
F
H,1,R1

R1 0.92197177

brh MP2= -2573.29528738 NIMAG= 0
Br
H,1,R1

R1 1.40660627

clh MP2= -460.31513005 NIMAG= 0
Cl
H,1,r1

r1=1.27483157

nch MP2= -93.25974985 NIMAG= 0
C
X,1,1.
N,1,r1,2,90.
H,1,r2,2,90.,3,180.,0

r1=1.16695694
r2=1.06463634

oh2 MP2= -76.32899232 NIMAG= 0
O
H,1,roh
H,1,roh,2,ahoh

roh=0.96133268
ahoh=104.10907946

hcch MP2= -77.16405740 NIMAG= 0
X
X,1,1.
C,1,r1,2,90.
C,1,r1,2,90.,3,180.,0
H,1,r2,2,90.,3,0.,0
H,1,r2,2,90.,3,180.,0

r1=0.60607914
r2=1.66782079

XB acids

clf MP2= -559.36182833 NIMAG= 0
F
Cl,1,r1

r1=1.63843742

clbr MP2= -3032.38347008 NIMAG= 0
Cl
Br,1,r1

r1=2.13811159

br2 MP2= -5145.37847619 NIMAG= 0
Br
Br,1,r1

r1=2.27860551

cl2 MP2= -919.38707879 NIMAG= 0
Cl
Cl,1,r1

r1=1.99871001

F_2 MP2= -199.29090711 NIMAG= 0
F
F,1,r1

r1=1.40135684

TB acids

geh3f MP2= -2177.14093842 NIMAG= 0
Ge,0.,0.0000000015,0.5109359231
F,0.,0.0000000015,2.2501473343
H,1.2544971355,0.7242842604,0.0816353147
H,-1.2544971355,0.7242842604,0.0816353147
H,0.,-1.4485685163,0.0816353147

sih3f MP2= -390.62206316 NIMAG= 0
Si,0.,0.0000000015,0.5557780523
F,0.,0.0000000015,2.1708879306
H,1.2121217237,0.699818805,0.0948822638
H,-1.2121217237,0.699818805,0.0948822638
H,0.,-1.3996376054,0.0948822638

f2co MP2= -312.63728452 NIMAG= 0
C
O,1,r1
F,1,r2,2,a2
F,1,r2,2,a2,3,180.,0

r1=1.17781394
r2=1.31649816
a2=126.2497747

co2 MP2= -188.32164060 NIMAG= 0
C
X,1,1.
O,1,r1,2,90.
O,1,r1,2,90.,3,180.,0

r1=1.17022433

ZB acids

ash2f MP2= -2335.37601117 NIMAG= 0
As
F,1,r1
H,1,r2,2,a2
H,1,r2,2,a2,3,d2,0

r1=1.75124575
r2=1.50609758
a2=95.48937657
d2=91.9672226

ph2f MP2= -441.82605456 NIMAG= 0
P
F,1,r1
H,1,r2,2,a2

H,1,r2,2,a2,3,d2,0

r1=1.62217546
r2=1.41599666
a2=97.67470617
d2=93.28270231

no2f MP2= -304.50987301 NIMAG= 0
N
F,1,r1
O,1,r2,2,a2
O,1,r2,2,a2,3,180.,0

r1=1.51841664
r2=1.17844359
a2=111.00723887

n2o MP2= -184.40679725 NIMAG= 0
N
X,1,1.
N,1,r1,2,90.
O,1,r2,2,90.,3,180.,0

r1=1.15537492
r2=1.18095902

YB acids
so3 MP2= -623.05943950 NIMAG= 0
S
X,1,1.
O,1,r1,2,90.
O,1,r1,2,90.,3,120.,0
O,1,r1,2,90.,3,-120.,0

r1=1.4451051

sef2 MP2= -2599.54587280 NIMAG= 0
Se
F,1,rsf
F,1,rsf,2,afsf

rsf=1.73058927
afsf=96.28663961

sf2 MP2= -597.13824319 NIMAG= 0
S
F,1,r1
F,1,r1,2,a1

r1=1.60529864
a1=98.15095064

so2_mp2 MP2= -547.96500959 NIMAG= 0
S
O,1,r1
O,1,r1,2,a1

r1=1.46355753
a1=118.80633544

seo2_mp2 MP2= -2550.32221616 NIMAG= 0
Se
O,1,r1
O,1,r1,2,a1

r1=1.62311933
a1=114.37553189

HB complexes with FH as Lewis Acid (LA)
fh_c3h6 MP2= -217.97459496 NIMAG= 0
F,-3.0219136879,0.,1.7447026812
H,-2.2151084356,0.,1.2788934516
C,-0.082039123,0.,0.9262270936
C,0.657946844,0.,-0.3798657875
C,-0.8431557542,0.,-0.3920655822
H,-0.0755889628,0.912130732,1.503903466
H,-0.0755889628,-0.912130732,1.503903466
H,1.1571205943,-0.9116354738,-0.6680638866
H,1.1571205943,0.9116354738,-0.6680638866
H,-1.3402130878,-0.912130732,-0.686489771
H,-1.3402130878,0.912130732,-0.686489771

fh_ch2o MP2= -214.67095039 NIMAG= 0
F,-0.7735200421,-1.558869925,0.
H,-0.6746964318,-0.6231794356,0.
O,-0.1139064859,0.9896294122,0.
C,1.1047984151,0.9985691275,0.
H,1.6810955975,0.0655319495,0.
H,1.6583439472,1.9440568715,0.

fh_co MP2= -213.49011856 NIMAG= 0
F
H,1,r1
X,2,1.,1,90.
C,2,rhb,3,90.,1,180.,0
O,2,r3,3,90.,1,180.,0

r1=0.92911551
rhb=2.05678022
r3=3.19254139

fh_h2cch2 MP2= -178.75368302 NIMAG= 0
F
H,1,r1
X,2,1.,1,90.
X,2,rhb,3,90.,1,180.,0
C,4,r3,2,90.,3,0.,0
C,4,r3,2,90.,3,180.,0
H,5,r4,4,a4,2,d4,0
H,5,r4,4,a4,2,-d4,0
H,6,r4,4,a4,2,d4,0
H,6,r4,4,a4,2,-d4,0

r1=0.93187181
rhb=2.12904889
r3=0.6683711
r4=1.0813083
a4=121.28292141
d4=90.18553592

fh_hcch MP2= -177.51272843 NIMAG= 0
F
H,1,r1
X,2,1.,1,90.
X,2,rhb,3,90.,1,180.,0
C,4,r3,2,90.,3,0.,0
C,4,r3,2,90.,3,180.,0
H,4,r4,2,a4,3,0.,0
H,4,r4,2,a4,3,180.,0

r1=0.93055659
rhb=2.12467627
r3=0.60674491
r4=1.66995715
a4=90.18871096

fh_n2 MP2= -209.71006157 NIMAG= 0
F
H,1,r1
X,2,1.,1,90.
N,2,rhb,3,90.,1,180.,0
N,2,r3,3,90.,1,180.,0

r1=0.9253417
rhb=2.05463754
r3=3.1678591

fh_nch MP2= -193.61331365 NIMAG= 0
F
H,1,r1
X,2,1.,1,90.
N,2,rhb,3,90.,1,180.,0
C,2,r3,3,90.,1,180.,0
H,2,r4,3,90.,1,180.,0

r1=0.93476867
rhb=1.83471805
r3=2.99798073
r4=4.06362442

fh_nh3 MP2= -156.82203686 NIMAG= 0
F
H,1,r1
X,2,1.,1,90.
N,2,rhb,3,90.,1,180.,0
H,4,r3,2,a3,3,0.,0
H,4,r3,2,a3,3,120.,0
H,4,r3,2,a3,3,-120.,0

r1=0.95720363
rhb=1.67882811
r3=1.01241736
a3=111.52709337

fh_oh2 MP2= -176.68415546 NIMAG= 0
F,0.8177960062,0.02887549,-0.0015948703
H,-0.1189302502,-0.0450858499,0.0003003372
O,-1.8212847631,-0.1128939846,-0.0013184212
H,-2.229172976,0.3020405166,0.7653704195
H,-2.2228937203,0.3176024016,-0.7627574652

fh_ph3 MP2= -443.01056795 NIMAG= 0
F
H,1,r1
X,2,1.,1,90.
P,2,rhb,3,90.,1,180.,0
H,4,r3,2,a3,3,0.,0
H,4,r3,2,a3,3,120.,0
H,4,r3,2,a3,3,-120.,0

r1=0.93410559
rhb=2.33567891
r3=1.40792329
a3=121.11428458

fh_sh2 MP2= -499.25855802 NIMAG= 0
F,1.5385745152,0.024423476,0.0002708338
H,0.6075981169,-0.0497568043,-0.0003201894
S,-1.6594472093,-0.1179840696,-0.0004515571
H,-1.7848597378,0.798431696,0.9656377957
H,-1.7868143822,0.799633605,-0.965136883

HB complexes with BrH as LA.
brh_c3h6 MP2= -2690.92854254 NIMAG= 0
Br,-3.4995298519,0.,2.0204545021
H,-2.2725982282,0.,1.3120851988
C,-0.0248332346,0.,0.8892162423
C,0.7203464359,0.,-0.4158922087
C,-0.7825004726,0.,-0.4231019092
H,-0.0210861,0.9112148213,1.4674886173
H,-0.0210861,-0.9112148213,1.4674886173
H,1.2203893826,-0.9109160851,-0.7045921386
H,1.2203893826,0.9109160851,-0.7045921386
H,-1.2814254724,-0.9112148213,-0.7154832104
H,-1.2814254724,0.9112148213,-0.7154832104

brh_ch2o MP2= -2687.62154076 NIMAG= 0
Br,-0.8281873394,-1.9484990364,0.
H,-0.7219088526,-0.5227992071,0.
O,-0.0818924776,1.2019152442,0.
C,1.1288703186,1.0697248894,0.
H,1.5919883512,0.0733260395,0.
H,1.7932449999,1.9420700705,0.

brh_co MP2= -2686.44196751 NIMAG= 0
Br
H,1,r1
X,2,1.,1,90.
C,2,rhb,3,90.,1,180.,0
O,2,r3,3,90.,1,180.,0

r1=1.41107335
rhb=2.35502889
r3=3.49237257

brh_h2cch2 MP2= -2651.70642392 NIMAG= 0
Br
H,1,r1
X,2,1.,1,90.
X,2,rhb,3,90.,1,180.,0
C,4,r3,2,90.,3,0.,0
C,4,r3,2,90.,3,180.,0
H,5,r4,4,a4,2,d4,0
H,5,r4,4,a4,2,-d4,0
H,6,r4,4,a4,2,d4,0

H,6,r4,4,a4,2,-d4,0

r1=1.41707942
rhb=2.30884684
r3=0.66791265
r4=1.08137705
a4=121.29811712
d4=90.11910321

brh_hcch MP2= -2650.46534279 NIMAG= 0
Br
H,1,r1
X,2,1.,1,90.
X,2,rhb,3,90.,1,180.,0
C,4,r3,2,90.,3,0.,0
C,4,r3,2,90.,3,180.,0
H,4,r4,2,a4,3,0.,0
H,4,r4,2,a4,3,180.,0

r1=1.41530045
rhb=2.30121446
r3=0.60671466
r4=1.66964063
a4=90.10966611

brh_n2 MP2= -2682.66317319 NIMAG= 0
Br
H,1,r1
X,2,1.,1,90.
N,2,rhb,3,90.,1,180.,0
N,2,r3,3,90.,1,180.,0

r1=1.40829685
rhb=2.34211163
r3=3.45603262

brh_nch MP2= -2666.56328483 NIMAG= 0
Br
H,1,r1
X,2,1.,1,90.
N,2,rhb,3,90.,1,180.,0
C,2,r3,3,90.,1,180.,0
H,2,r4,3,90.,1,180.,0

r1=1.41801562
rhb=2.05171364
r3=3.21708114
r4=4.2827802

brh_nh3 MP2= -2629.77018270 NIMAG= 0
Br
H,1,r1
X,2,1.,1,90.
N,2,rhb,3,90.,1,180.,0
H,4,r3,2,a3,3,0.,0
H,4,r3,2,a3,3,120.,0
H,4,r3,2,a3,3,-120.,0

r1=1.47543042
rhb=1.68679362
r3=1.01294726
a3=111.18675597

brh_oh2 MP2= -2649.63305874 NIMAG= 0
Br,1.3263162043,0.0001820227,0.
H,-0.0968549261,-0.0542384302,0.
O,-1.9892479294,-0.0854522491,0.
H,-2.4118867369,0.3213483989,0.7632940225
H,-2.4118867369,0.3213483989,-0.7632940225

brh_ph3 MP2= -2915.96259636 NIMAG= 0
Br
H,1,r1
X,2,1.,1,90.
P,2,rhb,3,90.,1,180.,0
H,4,r3,2,a3,3,0.,0
H,4,r3,2,a3,3,120.,0
H,4,r3,2,a3,3,-120.,0

r1=1.41970735
rhb=2.51941106
r3=1.40988236
a3=121.66887997

brh_sh2 MP2= -2972.21052513 NIMAG= 0
Br,1.9922334937,0.0085133346,0.
H,0.575447718,-0.0927943287,0.
S,-1.8584133976,-0.102247429,0.
H,-1.9035809732,0.8230456204,0.964548119
H,-1.9035809732,0.8230456204,-0.964548119

HB complexes with ClH as LA.
clh_c3h6 MP2= -577.94741124 NIMAG= 0
Cl,-3.3932378435,0.,1.9590867824
H,-2.2800003258,0.,1.3163588019
C,-0.0351532657,0.,0.8960169136
C,0.7091032477,0.,-0.409400951
C,-0.7935500423,0.,-0.4175648357

H,-0.0310885363,0.9119830855,1.4739512
H,-0.0310885363,-0.9119830855,1.4739512
H,1.2087145594,-0.9115432708,-0.6978516763
H,1.2087145594,0.9115432708,-0.6978516763
H,-1.2920234513,-0.9119830855,-0.7100521378
H,-1.2920234513,0.9119830855,-0.7100521378

clh_ch2o MP2= -574.64146168 NIMAG= 0
Cl,-0.8241695329,-1.8538087512,0.
H,-0.7238992687,-0.5603694347,0.
O,-0.0856326708,1.1544074724,0.
C,1.1283211559,1.0556729391,0.
H,1.6197024882,0.0734123893,0.
H,1.7677928284,1.9464233852,0.

clh_co MP2= -573.46190001 NIMAG= 0
Cl
H,1,r1
X,2,1.,1,90.
C,2,rhb,3,90.,1,180.,0
O,2,r3,3,90.,1,180.,0

r1=1.28008239
rhb=2.30119208
r3=3.4383378

clh_h2cch2 MP2= -538.72590467 NIMAG= 0
Cl
H,1,r1
X,2,1.,1,90.
X,2,rhb,3,90.,1,180.,0
C,4,r3,2,90.,3,0.,0
C,4,r3,2,90.,3,180.,0
H,5,r4,4,a4,2,d4,0
H,5,r4,4,a4,2,-d4,0
H,6,r4,4,a4,2,d4,0
H,6,r4,4,a4,2,-d4,0

r1=1.28536704
rhb=2.29659628
r3=0.66801317
r4=1.08136745
a4=121.29438859
d4=90.17941912

clh_hcch MP2= -537.48499312 NIMAG= 0
Cl
H,1,r1
X,2,1.,1,90.

X,2,rhb,3,90.,1,180.,0
C,4,r3,2,90.,3,0.,0
C,4,r3,2,90.,3,180.,0
H,4,r4,2,a4,3,0.,0
H,4,r4,2,a4,3,180.,0

r1=1.2837715
rhb=2.29181669
r3=0.6067046
r4=1.66967032
a4=90.1357729

clh_n2 MP2= -569.68287944 NIMAG= 0
Cl
H,1,r1
X,2,1.,1,90.
N,2,rhb,3,90.,1,180.,0
N,2,r3,3,90.,1,180.,0

r1=1.27708594
rhb=2.29741069
r3=3.41119829

clh_nch MP2= -553.58352474 NIMAG= 0
Cl
H,1,r1
X,2,1.,1,90.
N,2,rhb,3,90.,1,180.,0
C,2,r3,3,90.,1,180.,0
H,2,r4,3,90.,1,180.,0

r1=1.28740247
rhb=2.01456764
r3=3.17956143
r4=4.24523765

clh_nh3 MP2= -516.79036938 NIMAG= 0
Cl
H,1,r1
X,2,1.,1,90.
N,2,rhb,3,90.,1,180.,0
H,4,r3,2,a3,3,0.,0
H,4,r3,2,a3,3,120.,0
H,4,r3,2,a3,3,-120.,0

r1=1.3276727
rhb=1.73809169
r3=1.01286874
a3=111.4411735

clh_oh2 MP2= -536.65354273 NIMAG= 0
Cl,1.2028428978,0.0079046677,0.
H,-0.0884947363,-0.0567771812,0.
O,-1.9514805468,-0.0901103816,0.
H,-2.3694914004,0.3220840401,0.7628930766
H,-2.3694914004,0.3220840401,-0.7628930766

clh_ph3 MP2= -802.98237087 NIMAG= 0
Cl
H,1,r1
X,2,1.,1,90.
P,2,rhb,3,90.,1,180.,0
H,4,r3,2,a3,3,0.,0
H,4,r3,2,a3,3,120.,0
H,4,r3,2,a3,3,-120.,0

r1=1.28769729
rhb=2.50514892
r3=1.4097004
a3=121.61241138

clh_sh2 MP2= -859.23040820 NIMAG= 0
Cl,1.89042034,0.005143,0.
H,0.60504644,-0.08223579,0.
S,-1.81054853,-0.10284872,0.
H,-1.88731441,0.82044021,0.96448018
H,-1.88731441,0.82044021,-0.96448018

HB complexes with NCH as LA.
nch_c3h6 MP2= -210.89091200 NIMAG= 0
C,0.,0.,-2.9962841313
H,0.,0.,-1.9267193848
C,-0.7562972683,0.,0.3874304276
C,0.,0.,1.6864114709
C,0.7562972683,0.,0.3874304276
H,-1.2605703762,0.9119279334,0.1041346108
H,-1.2605703762,-0.9119279334,0.1041346108
H,0.,-0.9114459562,2.2633876486
H,0.,0.9114459562,2.2633876486
H,1.2605703762,-0.9119279334,0.1041346108
H,1.2605703762,0.9119279334,0.1041346108
N,0.,0.,-4.1638089933

nch_ch2o MP2= -207.58413608 NIMAG= 0
C,-1.1184491664,-2.0399507667,0.
H,-0.676971983,-1.0636425069,0.
O,0.21877723,0.8080898012,0.
C,1.3459535957,1.2624954352,0.
H,2.2298784943,0.610467394,0.

H,1.5233827505,2.3459067643,0.
N,-1.590455921,-3.1076281209,0.

nch_co MP2= -206.40613245 NIMAG= 0
C
H,1,r1
X,2,1.,1,90.
C,2,rhb,3,90.,1,180.,0
O,2,r3,3,90.,1,180.,0
N,2,r4,3,90.,1,0.,0

r1=1.06725723
rhb=2.4902353
r3=3.62745425
r4=2.23469048

nch_h2cch2 MP2= -171.66908419 NIMAG= 0
C
H,1,r1
X,2,1.,1,90.
X,2,rhb,3,90.,1,180.,0
C,4,r3,2,90.,3,0.,0
C,4,r3,2,90.,3,180.,0
H,5,r4,4,a4,2,d4,0
H,5,r4,4,a4,2,-d4,0
H,6,r4,4,a4,2,d4,0
H,6,r4,4,a4,2,-d4,0
N,2,rhn,3,90.,1,0.,0

r1=1.06922815
rhb=2.54005882
r3=0.66740569
r4=1.0814324
a4=121.31883116
d4=90.31340416
rhn=2.23672776

nch_hcch MP2= -170.42851209 NIMAG= 0
C
H,1,r1
X,2,1.,1,90.
X,2,rhb,3,90.,1,180.,0
C,4,r3,2,90.,3,0.,0
C,4,r3,2,90.,3,180.,0
H,4,r4,2,a4,3,0.,0
H,4,r4,2,a4,3,180.,0
N,2,rhn,3,90.,1,0.,0

r1=1.06873546
rhb=2.49788266

r3=0.60647287
r4=1.66940757
a4=90.4036798
rhn=2.2361276

nch_n2 MP2= -202.62737421 NIMAG= 0
C
H,1,r1
X,2,1.,1,90.
N,2,rhb,3,90.,1,180.,0
N,2,r3,3,90.,1,180.,0
N,2,rhn,3,90.,1,0.,0

r1=1.06579218
rhb=2.41148727
r3=3.52520283
rhn=2.23285662

nch_nch MP2= -186.52797679 NIMAG= 0
C
H,1,r1
X,2,1.,1,90.
N,2,rhb,3,90.,1,180.,0
C,2,r3,3,90.,1,180.,0
H,2,r4,3,90.,1,180.,0
N,2,rhn,3,90.,1,0.,0

r1=1.0710304
rhb=2.18643437
r3=3.35163877
r4=4.41747864
rhn=2.23861257

nch_nh3 MP2= -149.73114796 NIMAG= 0
C
H,1,r1
X,2,1.,1,90.
N,2,rhb,3,90.,1,180.,0
H,4,r3,2,a3,3,0.,0
H,4,r3,2,a3,3,120.,0
H,4,r3,2,a3,3,-120.,0
N,2,rhn,3,90.,1,0.,0

r1=1.08004845
rhb=2.10172987
r3=1.01309018
a3=112.25747627
rhn=2.24792663

nch_oh2 MP2= -169.59742215 NIMAG= 0
C,1.1547447813,-0.0176784621,0.0007128093
H,0.0845813222,0.0451308256,0.0008497036
O,-1.9558873458,0.1648599543,-0.0000515438
H,-2.5433365732,0.202015447,0.7605141771
H,-2.541187099,0.1955358082,-0.7625644984
N,2.3200874971,-0.0860451359,0.0005393522

nch_ph3 MP2= -435.92570770 NIMAG= 0
C
H,1,r1
X,2,1.,1,90.
P,2,rhb,3,90.,1,180.,0
H,4,r3,2,a3,3,0.,0
H,4,r3,2,a3,3,120.,0
H,4,r3,2,a3,3,-120.,0
N,2,rhn,3,90.,1,0.,0

r1=1.06949598
rhb=2.79382228
r3=1.41011932
a3=121.89303975
rhn=2.23705586

nch_sh2 MP2= -492.17364430 NIMAG= 0
C,1.8431842668,-0.0533846161,0.0001009456
H,0.7734881194,-0.0827759992,-0.0006225517
S,-1.8962684296,-0.0705749637,-0.0016081756
H,-2.0525839155,0.8395634552,0.9653092865
H,-2.0538315313,0.8440757941,-0.9640507402
N,3.0101478025,-0.0170652243,0.0008712354

HB complexes with OH_2 as LA.
h2o_c3h6 MP2= -193.95953533 NIMAG= 0
O,-0.1773367816,2.589663768,0.
H,-0.0100970191,1.6390544226,0.
C,-0.732003558,-0.599640684,0.
C,0.0063431176,-1.9079847616,0.
C,0.7813434751,-0.6190885779,0.
H,-1.2291323747,-0.3035759276,0.9112572437
H,-1.2291323747,-0.3035759276,-0.9112572437
H,-0.0001565789,-2.4853470227,-0.9112423633
H,-0.0001565789,-2.4853470227,0.9112423633
H,1.2881394178,-0.3429631097,-0.9125188954
H,1.2881394178,-0.3429631097,0.9125188954
H,0.698508857,2.9854722326,0.

h2o_ch2o MP2= -190.65429023 NIMAG= 0
O,-0.6694620392,-1.5395863789,0.

H,-0.8410945166,-0.5859857107,0.
O,-0.1248061158,1.2510366612,0.
C,1.0686367146,1.011021876,0.
H,1.4415541833,-0.0213350508,0.
H,1.8091403985,1.8217149227,0.
H,-1.5390558786,-1.9470499153,0.

h2o_co MP2= -189.47507759 NIMAG= 0
O,0.0232090283,0.,-0.1103883994
H,0.179559469,0.,0.8403482732
C,0.0806152511,0.,3.1754590613
O,-0.0849016476,0.,4.3006418962
H,0.9029372165,0.,-0.4970184445

h2o_h2cch2 MP2= -154.73844988 NIMAG= 0
O,0.,0.1327787937,-0.1516961336
H,0.,-0.0786390442,0.7900258465
C,0.6676860969,0.0178636836,3.1545329219
C,-0.6676860969,0.0178636836,3.1545329219
H,1.2294207237,0.9103283899,2.9153666252
H,1.2296103964,-0.8735180142,3.3970511782
H,-1.2296103964,-0.8735180142,3.3970511782
H,-1.2294207237,0.9103283899,2.9153666252
H,0.,-0.7234624674,-0.5883652319

h2o_hcch MP2= -153.49785578 NIMAG= 0
O,0.,-0.0193158489,-0.1640822417
H,0.,-0.0206730989,0.8007921989
C,0.6065664969,-0.0026265255,3.1486918976
C,-0.6065664969,-0.0026265255,3.1486918976
H,1.6692825851,0.0023361987,3.1502315511
H,-1.6692825851,0.0023361987,3.1502315511
H,0.,-0.9515655457,-0.3987959119

h2o_n2 MP2= -185.69629795 NIMAG= 0
O,0.0172429191,0.,-0.0895105113
H,0.2178405497,0.,0.8515182966
N,0.1004140457,0.,3.1664400439
N,-0.1169848797,0.,4.2587106217
H,0.8782903468,0.,-0.5164183973

h2o_nch MP2= -169.59574819 NIMAG= 0
O,-0.0597552849,0.,-0.1322289719
H,0.063320241,0.,0.8263910189
N,0.1309964212,0.,2.9137580046
C,-0.0280901658,0.,4.0680748519
H,-0.1753100217,0.,5.1233174068
H,0.8338084842,0.,-0.4845165795

h2o_nh3 MP2= -132.80022060 NIMAG= 0
O,0.032764493,0.,-0.1563983244
H,-0.103725514,0.,0.8090367155
N,-0.0331593354,0.,2.7649196434
H,0.9619211997,0.,2.9538030231
H,-0.4199215662,0.8142695303,3.2255584151
H,-0.4199215662,-0.8142695303,3.2255584151
H,-0.8525183565,0.,-0.5283780512

h2o_oh2 MP2= -152.66624078 NIMAG= 0
O,0.9390160235,0.0199285438,0.0237854182
H,-0.0191087769,-0.1218441481,0.0170976713
O,-1.9644741166,-0.1258243038,0.0025578235
H,-2.3174482169,0.3577615857,0.7558963934
H,-2.3084030972,0.3399037535,-0.766054659
H,1.3126395115,-0.8647417858,0.0362050849

h2o_ph3 MP2= -418.99478274 NIMAG= 0
O,0.0838353504,0.,-0.1645941168
H,-0.0803045345,0.,0.7871454572
P,-0.0394874222,0.,3.4015606155
H,1.2608173374,0.,3.9464840623
H,-0.5128943303,1.0373996266,4.2313349196
H,-0.5128943303,-1.0373996266,4.2313349196
H,-0.794056,0.,-0.5558737559

h2o_sh2 MP2= -475.24307387 NIMAG= 0
O,1.5929309852,0.0307345685,-0.0023009613
H,0.6502260216,-0.181099317,-0.0041688081
S,-1.8613763358,-0.1020038633,-0.0011871402
H,-1.7752596671,0.8178949926,0.9653259374
H,-1.7823946622,0.8227285003,-0.9636791507
H,2.0291417938,-0.8256912627,-0.0045001035

HB complexes with HCCH as LA.
hcch_c3h6 MP2= -194.79361964 NIMAG= 0
C,0.,0.,-3.0554337936
H,0.,0.,-1.9906758254
C,-0.7544469289,0.,0.4304120129
C,0.,0.,1.731593328
C,0.7544469289,0.,0.4304120129
H,-1.2568597579,0.9112242994,0.1428207185
H,-1.2568597579,-0.9112242994,0.1428207185
H,0.,-0.9110158235,2.3094915289
H,0.,0.9110158235,2.3094915289
H,1.2568597579,-0.9112242994,0.1428207185
H,1.2568597579,0.9112242994,0.1428207185
C,0.,0.,-4.2686319111

H,0.,0.,-5.330771767

hcch_ch2o MP2= -191.48597275 NIMAG= 0
C,-1.1366689588,-1.6107342383,0.
H,-1.1592803107,-0.5437871811,0.
O,-0.0155702032,1.3649092643,0.
C,1.1125852916,0.9134035678,0.
H,1.2967892127,-0.171038323,0.
H,1.9925168325,1.5715717481,0.
C,-1.093716488,-2.8234913659,0.
H,-1.0645403761,-3.8850954716,0.

hcch_co MP2= -190.30908154 NIMAG= 0
C
H,1,r1
X,2,1.,1,90.
C,2,rhb,3,90.,1,180.,0
O,2,r3,3,90.,1,180.,0
C,2,r4,3,90.,1,0.,0
H,2,r5,3,90.,1,0.,0

r1=1.06340467
rhb=2.60031902
r3=3.73827533
r4=2.27625971
r5=3.33832867

hcch_h2cch2 MP2= -155.57182035 NIMAG= 0
C
H,1,r1
X,2,1.,1,90.
X,2,rhb,3,90.,1,180.,0
C,4,r3,2,90.,3,0.,0
C,4,r3,2,90.,3,180.,0
H,5,r4,4,a4,2,d4,0
H,5,r4,4,a4,2,-d4,0
H,6,r4,4,a4,2,d4,0
H,6,r4,4,a4,2,-d4,0
C,2,rhn,3,90.,1,0.,0
H,2,rhh,3,90.,1,0.,0

r1=1.06460837
rhb=2.64489197
r3=0.66708366
r4=1.08126873
a4=121.315637
d4=90.03223472
rhn=2.27773438
rhh=3.33976491

hcch_hcch MP2= -154.33126151 NIMAG= 0
C
H,1,r1
X,2,1.,1,90.
X,2,rhb,3,90.,1,180.,0
C,4,r3,2,90.,3,0.,0
C,4,r3,2,90.,3,180.,0
H,4,r4,2,a4,3,0.,0
H,4,r4,2,a4,3,180.,0
C,2,rhn,3,90.,1,0.,0
H,2,rhh,3,90.,1,0.,0

r1=1.06443076
rhb=2.60170162
r3=0.60634341
r4=1.66884045
a4=90.06745592
rhn=2.2775024
rhh=3.33955103

hcch_n2 MP2= -186.53088120 NIMAG= 0
C
H,1,r1
X,2,1.,1,90.
N,2,rhb,3,90.,1,180.,0
N,2,r3,3,90.,1,180.,0
C,2,rhn,3,90.,1,0.,0
H,2,rhh,3,90.,1,0.,0

r1=1.06241443
rhb=2.49849361
r3=3.61242067
rhn=2.27501413
rhh=3.33717446

hcch_nch MP2= -170.42872418 NIMAG= 0
C
H,1,r1
X,2,1.,1,90.
N,2,rhb,3,90.,1,180.,0
C,2,r3,3,90.,1,180.,0
H,2,r4,3,90.,1,180.,0
C,2,rhn,3,90.,1,0.,0
H,2,rhh,3,90.,1,0.,0

r1=1.06593671
rhb=2.32094968
r3=3.48706834
r4=4.55250826
rhn=2.27931545

rhh=3.34142218

hcch_nh3 MP2= -133.63090087 NIMAG= 0
C
H,1,r1
X,2,1.,1,90.
N,2,rhb,3,90.,1,180.,0
H,4,r3,2,a3,3,0.,0
H,4,r3,2,a3,3,120.,0
H,4,r3,2,a3,3,-120.,0
C,2,rhn,3,90.,1,0.,0
H,2,rhh,3,90.,1,0.,0

r1=1.07098623
rhb=2.25873144
r3=1.01271129
a3=112.13981235
rhn=2.28519723
rhh=3.34726806

hcch_oh2 MP2= -153.49809380 NIMAG= 0
C,-0.01219986,1.04766174,0.
H,-0.03058796,-0.0188389,0.
O,-0.04659835,-2.20723016,0.
H,0.18524722,-2.74775665,0.7608844
H,0.18524722,-2.74775665,-0.7608844
C,0.01231162,2.26083752,0.
H,0.03180715,3.3226606,0.

hcch_ph3 MP2= -419.82818949 NIMAG= 0
C
H,1,r1
X,2,1.,1,90.
P,2,rhb,3,90.,1,180.,0
H,4,r3,2,a3,3,0.,0
H,4,r3,2,a3,3,120.,0
H,4,r3,2,a3,3,-120.,0
C,2,rhn,3,90.,1,0.,0
H,2,rhh,3,90.,1,0.,0

r1=1.06475892
rhb=2.92555221
r3=1.41133495
a3=122.2677193
rhn=2.2779491
rhh=3.33974996

hcch_sh2 MP2= -476.07612169 NIMAG= 0
C,1.86712938,-0.063456437,-0.0007042037

H,0.8050689275,-0.1448963745,-0.0007043134
S,-1.9893434909,-0.0925450005,-0.0002128219
H,-2.0429262702,0.8315750254,0.9642180344
H,-2.0421687907,0.8330296478,-0.963291106
C,3.0766245962,0.0309466303,0.0001262363
H,4.1353992727,0.1129429358,0.0005681742

XB complexes with FCl as LA.

fcl_c3h6 MP2= -676.99444358 NIMAG= 0
F,-4.1558902693,0.,2.3994043658
Cl,-2.7259040817,0.,1.5738014554
C,0.0996625211,0.,0.819803635
C,0.8425033474,0.,-0.4864195344
C,-0.6601395135,0.,-0.4962120926
H,0.1045293308,0.9115975011,1.397071936
H,0.1045293308,-0.9115975011,1.397071936
H,1.3421732607,-0.9116065459,-0.7749040934
H,1.3421732607,0.9116065459,-0.7749040934
H,-1.1576351221,-0.9115975011,-0.7890610239
H,-1.1576351221,0.9115975011,-0.7890610239

fcl_ch2o MP2= -673.68854369 NIMAG= 0
F,-1.4748001597,-2.4059774252,0.
Cl,-0.8908835759,-0.8514875758,0.
O,0.1190613448,1.3608532375,0.
C,1.3329863752,1.2659696764,0.
H,1.8280973858,0.2861406354,0.
H,1.9676536298,2.1602394518,0.

fcl_co MP2= -672.51018986 NIMAG= 0
F
Cl,1,r1
X,2,1.,1,90.
C,2,rhb,3,90.,1,180.,0
O,2,r3,3,90.,1,180.,0

r1=1.65127076
rhb=2.66110144
r3=3.79799892

fcl_h2cch2 MP2= -637.77743539 NIMAG= 0
F
Cl,1,r1
X,2,1.,1,90.
X,2,rhb,3,90.,1,180.,0
C,4,r3,2,90.,3,0.,0
C,4,r3,2,90.,3,180.,0
H,5,r4,4,a4,2,d4,0
H,5,r4,4,a4,2,-d4,0

H,6,r4,4,a4,2,d4,0
H,6,r4,4,a4,2,-d4,0

r1=1.68736661
rhb=2.50979579
r3=0.6722465
r4=1.08071665
a4=121.18689166
d4=90.76625077

fcl_hcch MP2= -636.53375856 NIMAG= 0
F
Cl,1,r1
X,2,1.,1,90.
X,2,rhb,3,90.,1,180.,0
C,4,r3,2,90.,3,0.,0
C,4,r3,2,90.,3,180.,0
H,4,r4,2,a4,3,0.,0
H,4,r4,2,a4,3,180.,0

r1=1.65940748
rhb=2.72770772
r3=0.60756389
r4=1.67045164
a4=90.52271138

fcl_n2 MP2= -668.73026637 NIMAG= 0
F
Cl,1,r1
X,2,1.,1,90.
N,2,rhb,3,90.,1,180.,0
N,2,r3,3,90.,1,180.,0

r1=1.64210542
rhb=2.80294372
r3=3.91695545

fcl_nch MP2= -652.63084016 NIMAG= 0
F
Cl,1,r1
X,2,1.,1,90.
N,2,rhb,3,90.,1,180.,0
C,2,r3,3,90.,1,180.,0
H,2,r4,3,90.,1,180.,0

r1=1.65568904
rhb=2.54144619
r3=3.70634624
r4=4.77190894

fcl_nh3 MP2= -615.84120762 NIMAG= 0
F
Cl,1,r1
X,2,1.,1,90.
N,2,rhb,3,90.,1,180.,0
H,4,r3,2,a3,3,0.,0
H,4,r3,2,a3,3,120.,0
H,4,r3,2,a3,3,-120.,0

r1=1.714061
rhb=2.233069
r3=1.01226772
a3=110.1552828

fcl_oh2 MP2= -635.69975104 NIMAG= 0
F,1.8521796224,-0.0296887564,-0.0022938264
Cl,0.196781258,-0.0653780035,0.0013504885
O,-2.3192804161,-0.1208031406,0.0062590909
H,-2.6572455843,0.375295106,0.7593350408
H,-2.6578212121,0.3466886378,-0.7646507939

fcl_ph3 MP2= -902.04251903 NIMAG= 0
F
Cl,1,r1
X,2,1.,1,90.
P,2,rhb,3,90.,1,180.,0
H,4,r3,2,a3,3,0.,0
H,4,r3,2,a3,3,120.,0
H,4,r3,2,a3,3,-120.,0

r1=1.8508506
rhb=2.18284465
r3=1.40140984
a3=116.7154972

fcl_sh2 MP2= -958.28047629 NIMAG= 0
F,2.4020228994,-0.088594387,0.0006158197
Cl,0.7204608602,-0.0824188569,-0.002370275
S,-2.0002617204,-0.0697616937,-0.007640341
H,-2.110654673,0.836384689,0.9701498014
H,-2.1060740605,0.86283797,-0.9607550051

XB complexes with ClBr as LA.
clbr_c3h6 MP2= -3150.01785077 NIMAG= 0
Cl,-4.6383146236,0.,2.6779321966
Br,-2.7738606632,0.,1.6014892006
C,0.1566075184,0.,0.7860952327
C,0.9006041314,0.,-0.5199640377

C,-0.6024746821,0.,-0.5286737057
H,0.1607033781,0.911793203,1.3633502399
H,0.1607033781,-0.911793203,1.3633502399
H,1.4000226564,-0.9117636242,-0.8083034576
H,1.4000226564,0.9117636242,-0.8083034576
H,-1.100344253,-0.911793203,-0.8208483278
H,-1.100344253,0.911793203,-0.8208483278

clbr_ch2o MP2= -3146.70988604 NIMAG= 0
Cl,-1.7221089876,-2.8543773823,0.
Br,-0.9568809127,-0.8358959766,0.
O,0.2019604731,1.5210952832,0.
C,1.410212835,1.3738274033,0.
H,1.8628737197,0.3729440799,0.
H,2.0860578724,2.2381445926,0.

clbr_co MP2= -3145.53149803 NIMAG= 0
Cl
Br,1,r1
X,2,1.,1,90.
C,2,rhb,3,90.,1,180.,0
O,2,r3,3,90.,1,180.,0

r1=2.14970462
rhb=2.89862094
r3=4.03598931

clbr_h2cch2 MP2= -3110.79915384 NIMAG= 0
Cl
Br,1,r1
X,2,1.,1,90.
X,2,rhb,3,90.,1,180.,0
C,4,r3,2,90.,3,0.,0
C,4,r3,2,90.,3,180.,0
H,5,r4,4,a4,2,d4,0
H,5,r4,4,a4,2,-d4,0
H,6,r4,4,a4,2,d4,0
H,6,r4,4,a4,2,-d4,0

r1=2.18033702
rhb=2.74212946
r3=0.67108024
r4=1.08107835
a4=121.20318056
d4=90.45368237

clbr_hcch MP2= -3109.55571003 NIMAG= 0
Cl
Br,1,r1

X,2,1.,1,90.
X,2,rhb,3,90.,1,180.,0
C,4,r3,2,90.,3,0.,0
C,4,r3,2,90.,3,180.,0
H,4,r4,2,a4,3,0.,0
H,4,r4,2,a4,3,180.,0

r1=2.15854038
rhb=2.90706058
r3=0.60749035
r4=1.67043073
a4=90.40740443

clbr_n2 MP2= -3141.75215627 NIMAG= 0
Cl
Br,1,r1
X,2,1.,1,90.
N,2,rhb,3,90.,1,180.,0
N,2,r3,3,90.,1,180.,0

r1=2.14264304
rhb=2.97872131
r3=4.09291264

clbr_nch MP2= -3125.65238008 NIMAG= 0
Cl
Br,1,r1
X,2,1.,1,90.
N,2,rhb,3,90.,1,180.,0
C,2,r3,3,90.,1,180.,0
H,2,r4,3,90.,1,180.,0

r1=2.15551009
rhb=2.72208327
r3=3.88777103
r4=4.95356283

clbr_nh3 MP2= -3088.86007270 NIMAG= 0
Cl
Br,1,r1
X,2,1.,1,90.
N,2,rhb,3,90.,1,180.,0
H,4,r3,2,a3,3,0.,0
H,4,r3,2,a3,3,120.,0
H,4,r3,2,a3,3,-120.,0

r1=2.20281796
rhb=2.46906228
r3=1.0126311
a3=110.7595784

clbr_oh2 MP2= -3108.72089915 NIMAG= 0
Cl,2.3739348901,-0.0558293231,0.0003775627
Br,0.2194543278,-0.0724313884,0.0008917601
O,-2.4782408765,-0.0928297748,0.0054111865
H,-2.8477744455,0.3802414747,0.7583239798
H,-2.852760228,0.3469628547,-0.7650044894

clbr_ph3 MP2= -3375.05674853 NIMAG= 0
Cl
Br,1,r1
X,2,1.,1,90.
P,2,rhb,3,90.,1,180.,0
H,4,r3,2,a3,3,0.,0
H,4,r3,2,a3,3,120.,0
H,4,r3,2,a3,3,-120.,0

r1=2.24482976
rhb=2.65905645
r3=1.40588044
a3=119.37317054

clbr_sh2 MP2= -3431.30142776 NIMAG= 0
Cl,2.921495485,-0.1369548113,-0.00018186
Br,0.7478239578,-0.1136045323,-0.0030778664
S,-2.2233964808,-0.047026982,-0.0062078515
H,-2.2710330905,0.8665608337,0.9696867875
H,-2.2693965658,0.8894732135,-0.9602192095

XB complexes with Br_2 as LA.
br2_c3h6 MP2= -5263.01275137 NIMAG= 0
Br,-4.7701341432,0.,2.7540382317
Br,-2.7861795395,0.,1.6086015072
C,0.1714132456,0.,0.7762772007
C,0.91694684,0.,-0.529399505
C,-0.5865691534,0.,-0.5365868256
H,0.1741670561,0.9116274056,1.3538892717
H,0.1741670561,-0.9116274056,1.3538892717
H,1.4165071018,-0.9117288956,-0.8178207566
H,1.4165071018,0.9117288956,-0.8178207566
H,-1.0854189752,-0.9116274056,-0.8277777309
H,-1.0854189752,0.9116274056,-0.8277777309

br2_ch2o MP2= -5259.70392888 NIMAG= 0
Br,-1.7618429841,-2.9740726173,0.
Br,-0.9732891986,-0.8187363541,0.
O,0.2213944285,1.5872757296,0.
C,1.4232787088,1.3991641033,0.
H,1.8422225048,0.3832277001,0.

H,2.1303515405,2.2388794386,0.

br2_co MP2= -5258.52584909 NIMAG= 0
Br
Br,1,r1
X,2,1.,1,90.
C,2,rhb,3,90.,1,180.,0
O,2,r3,3,90.,1,180.,0

r1=2.28717454
rhb=2.98699196
r3=4.12470205

br2_h2cch2 MP2= -5223.79313927 NIMAG= 0
Br
Br,1,r1
X,2,1.,1,90.
X,2,rhb,3,90.,1,180.,0
C,4,r3,2,90.,3,0.,0
C,4,r3,2,90.,3,180.,0
H,5,r4,4,a4,2,d4,0
H,5,r4,4,a4,2,-d4,0
H,6,r4,4,a4,2,d4,0
H,6,r4,4,a4,2,-d4,0

r1=2.31393702
rhb=2.81476708
r3=0.67035066
r4=1.08113438
a4=121.21417692
d4=90.34296159

br2_hcch MP2= -5222.55007313 NIMAG= 0
Br
Br,1,r1
X,2,1.,1,90.
X,2,rhb,3,90.,1,180.,0
C,4,r3,2,90.,3,0.,0
C,4,r3,2,90.,3,180.,0
H,4,r4,2,a4,3,0.,0
H,4,r4,2,a4,3,180.,0

r1=2.29574899
rhb=2.96516749
r3=0.60731997
r4=1.67013311
a4=90.330972

br2_n2 MP2= -5254.74693700 NIMAG= 0

Br
Br,1,r1
X,2,1.,1,90.
N,2,rhb,3,90.,1,180.,0
N,2,r3,3,90.,1,180.,0

r1=2.2819367
rhb=3.03831676
r3=4.15255529

br2_nch MP2= -5238.64634281 NIMAG= 0
Br
Br,1,r1
X,2,1.,1,90.
N,2,rhb,3,90.,1,180.,0
C,2,r3,3,90.,1,180.,0
H,2,r4,3,90.,1,180.,0

r1=2.29211177
rhb=2.78887038
r3=3.95496062
r4=5.020644

br2_nh3 MP2= -5201.85272142 NIMAG= 0
Br
Br,1,r1
X,2,1.,1,90.
N,2,rhb,3,90.,1,180.,0
H,4,r3,2,a3,3,0.,0
H,4,r3,2,a3,3,120.,0
H,4,r3,2,a3,3,-120.,0

r1=2.33234293
rhb=2.53805106
r3=1.01252703
a3=110.8961989

br2_oh2 MP2= -5221.71484739 NIMAG= 0
Br,1.7024568281,0.0105979409,0.
Br,-0.5888553341,-0.0271340903,0.
O,-3.3454027973,-0.0637603509,0.
H,-3.7211181886,0.3903657864,0.7611839187
H,-3.7211181886,0.3903657864,-0.7611839187

br2_ph3 MP2= -5488.04964841 NIMAG= 0
Br
Br,1,r1
X,2,1.,1,90.
P,2,rhb,3,90.,1,180.,0

H,4,r3,2,a3,3,0.,0
H,4,r3,2,a3,3,120.,0
H,4,r3,2,a3,3,-120.,0

r1=2.34486864
rhb=2.83570903
r3=1.40790653
a3=120.40984932

br2_sh2 MP2= -5544.29539551 NIMAG= 0
Br,3.0536637724,-0.1414478613,0.
Br,0.74597777,-0.126907049,0.
S,-2.2977933969,-0.0515978189,0.
H,-2.3121814674,0.8747439663,0.9645999635
H,-2.3121814674,0.8747439663,-0.9645999635

XB complexes with Cl_2 as LA.
cl2_c3h6 MP2= -1037.01797887 NIMAG= 0
Cl,-4.5482079098,0.,2.6259090612
Cl,-2.8097770342,0.,1.6222255271
C,0.1487671242,0.,0.7871255893
C,0.8966427563,0.,-0.5176769367
C,-0.6072871942,0.,-0.5223989034
H,0.1502551091,0.9114146159,1.3656005757
H,0.1502551091,-0.9114146159,1.3656005757
H,1.3970713402,-0.9114017044,-0.8065995144
H,1.3970713402,0.9114017044,-0.8065995144
H,-1.1075172355,-0.9114146159,-0.8129250293
H,-1.1075172355,0.9114146159,-0.8129250293

cl2_ch2o MP2= -1033.70959612 NIMAG= 0
Cl,-1.6142845414,-2.7706539697,0.
Cl,-0.979847742,-0.8630504521,0.
O,0.1895206289,1.5707227643,0.
C,1.3863284682,1.3594221603,0.
H,1.787638617,0.3357404668,0.
H,2.1127595692,2.1835570305,0.

cl2_co MP2= -1032.53268343 NIMAG= 0
Cl
Cl,1,r1
X,2,1.,1,90.
C,2,rhb,3,90.,1,180.,0
O,2,r3,3,90.,1,180.,0

r1=2.00375505
rhb=3.03551383
r3=4.17359798

cl2_h2cch2 MP2= -997.79772732 NIMAG= 0
Cl
Cl,1,r1
X,2,1.,1,90.
X,2,rhb,3,90.,1,180.,0
C,4,r3,2,90.,3,0.,0
C,4,r3,2,90.,3,180.,0
H,5,r4,4,a4,2,d4,0
H,5,r4,4,a4,2,-d4,0
H,6,r4,4,a4,2,d4,0
H,6,r4,4,a4,2,-d4,0

r1=2.01891704
rhb=2.8922183
r3=0.66861896
r4=1.08116817
a4=121.27332434
d4=90.08805508

cl2_hcch MP2= -996.55599495 NIMAG= 0
Cl
Cl,1,r1
X,2,1.,1,90.
X,2,rhb,3,90.,1,180.,0
C,4,r3,2,90.,3,0.,0
C,4,r3,2,90.,3,180.,0
H,4,r4,2,a4,3,0.,0
H,4,r4,2,a4,3,180.,0

r1=2.00968484
rhb=2.99945509
r3=0.60685404
r4=1.66933948
a4=90.13847141

cl2_n2 MP2= -1028.75433916 NIMAG= 0
Cl
Cl,1,r1
X,2,1.,1,90.
N,2,rhb,3,90.,1,180.,0
N,2,r3,3,90.,1,180.,0

r1=2.00160615
rhb=3.01808149
r3=4.13224305

cl2_nch MP2= -1012.65225269 NIMAG= 0
Cl
Cl,1,r1

X,2,1.,1,90.
N,2,rhb,3,90.,1,180.,0
C,2,r3,3,90.,1,180.,0
H,2,r4,3,90.,1,180.,0

r1=2.0075415
rhb=2.82201601
r3=3.98844221
r4=5.05380255

cl2_nh3 MP2= -975.85634482 NIMAG= 0
Cl
Cl,1,r1
X,2,1.,1,90.
N,2,rhb,3,90.,1,180.,0
H,4,r3,2,a3,3,0.,0
H,4,r3,2,a3,3,120.,0
H,4,r3,2,a3,3,-120.,0

r1=2.03365915
rhb=2.59183518
r3=1.01243456
a3=111.37679814

cl2_oh2 MP2= -995.72110781 NIMAG= 0
Cl,2.3016950541,-0.0554474843,-0.0027285506
Cl,0.2936565321,-0.0753519428,0.0032892135
O,-2.4806867579,-0.0945003294,0.0057299512
H,-2.8476874047,0.3822398791,0.7567788716
H,-2.8523637558,0.3491737207,-0.7630694858

cl2_ph3 MP2= -1262.05385212 NIMAG= 0
Cl
Cl,1,r1
X,2,1.,1,90.
P,2,rhb,3,90.,1,180.,0
H,4,r3,2,a3,3,0.,0
H,4,r3,2,a3,3,120.,0
H,4,r3,2,a3,3,-120.,0

r1=2.02461019
rhb=3.05029103
r3=1.41031988
a3=121.64861689

cl2_sh2 MP2= -1318.30106828 NIMAG= 0
Cl,2.8588522505,-0.1331798498,0.0003879121
Cl,0.8424911141,-0.1229298729,-0.0027420954
S,-2.2568463683,-0.0460101839,-0.0071315622

H,-2.2719337245,0.8678934841,0.9686760906
H,-2.2670699661,0.892674144,-0.9591903451

XB complexes with F_2 as LA.

ff_c3h6 MP2= -316.91903395 NIMAG= 0
F,-3.9454303751,0.,2.2778952892
F,-2.726705296,0.,1.5742640367
C,0.0746397303,0.,0.8273122272
C,0.8253947919,0.,-0.4765419053
C,-0.6791535405,0.,-0.4782960161
H,0.0743305202,0.9106955489,1.4060538259
H,0.0743305202,-0.9106955489,1.4060538259
H,1.3263638738,-0.9107730695,-0.7657765396
H,1.3263638738,0.9107730695,-0.7657765396
H,-1.1805130722,-0.9106955489,-0.7673990317
H,-1.1805130722,0.9106955489,-0.7673990317

ff_ch2o MP2= -313.61007741 NIMAG= 0
F,-1.1994183404,-2.2635647276,0.
F,-0.9105111155,-0.8853934625,0.
O,0.0830831709,1.5219018826,0.
C,1.2629876218,1.2378143737,0.
H,1.603163122,0.1917422394,0.
H,2.0428105412,2.0132376944,0.

ff_co MP2= -312.43483931 NIMAG= 0
F,0.,0.,-0.1046475904
F,0.,0.,1.3001710277
C,0.,0.,4.1794838328
O,0.,0.,5.3178827298

ff_h2cch2 MP2= -277.69816438 NIMAG= 0
F
F,1,r1
X,2,1.,1,90.
X,2,rhb,3,90.,1,180.,0
C,4,r3,2,90.,3,0.,0
C,4,r3,2,90.,3,180.,0
H,5,r4,4,a4,2,d4,0
H,5,r4,4,a4,2,-d4,0
H,6,r4,4,a4,2,d4,0
H,6,r4,4,a4,2,-d4,0

r1=1.41345289
rhb=2.76806951
r3=0.66733773
r4=1.081009
a4=121.29691877
d4=90.00026839

ff_hcch MP2= -276.45717986 NIMAG= 0
F
F,1,r1
X,2,1.,1,90.
X,2,rhb,3,90.,1,180.,0
C,4,r3,2,90.,3,0.,0
C,4,r3,2,90.,3,180.,0
H,4,r4,2,a4,3,0.,0
H,4,r4,2,a4,3,180.,0

r1=1.4085862
rhb=2.85528888
r3=0.60637066
r4=1.66839035
a4=89.99099456

ff_n2 MP2= -308.65705700 NIMAG= 0
F
F,1,r1
X,2,1.,1,90.
N,2,rhb,3,90.,1,180.,0
N,2,r3,3,90.,1,180.,0

r1=1.40354926
rhb=2.82297114
r3=3.93706528

ff_nch MP2= -292.55309406 NIMAG= 0
F
F,1,r1
X,2,1.,1,90.
N,2,rhb,3,90.,1,180.,0
C,2,r3,3,90.,1,180.,0
H,2,r4,3,90.,1,180.,0

r1=1.40682961
rhb=2.70616577
r3=3.87298762
r4=4.93804325

ff_nh3 MP2= -255.75468851 NIMAG= 0
F
F,1,r1
X,2,1.,1,90.
N,2,rhb,3,90.,1,180.,0
H,4,r3,2,a3,3,0.,0
H,4,r3,2,a3,3,120.,0
H,4,r3,2,a3,3,-120.,0

r1=1.41629674
rhb=2.59357104
r3=1.01226183
a3=111.93157218

ff_oh2 MP2= -275.62217342 NIMAG= 0
F,1.7820075601,-0.065417344,0.0037161016
F,0.3753698684,-0.046653826,-0.0007681207
O,-2.2730710311,-0.039011243,-0.0005131851
H,-2.7244939109,0.3430606585,0.7577294241
H,-2.7451988187,0.3141355977,-0.76016422

ff_ph3 MP2= -541.95432363 NIMAG= 0
F
F,1,r1
X,2,1.,1,90.
P,2,rhb,3,90.,1,180.,0
H,4,r3,2,a3,3,0.,0
H,4,r3,2,a3,3,120.,0
H,4,r3,2,a3,3,-120.,0

r1=1.41188267
rhb=3.04815812
r3=1.41184501
a3=122.39260167

ff_sh2 MP2= -598.20185351 NIMAG= 0
F,2.3465860899,-0.0970474283,-0.0031218846
F,0.9360221066,-0.1000931094,-0.0018230594
S,-2.0848601342,-0.064286329,-0.0074580254
H,-2.1575055425,0.8449477902,0.9693603632
H,-2.1347492141,0.8749267978,-0.9569573938

TB complexes with GeH_3F as LA.
geh3f_c3h6 MP2= -2294.77331729 NIMAG= 0
Ge,-0.0396349691,1.5597928315,-0.0000000009
F,-0.1790868839,3.2990232628,-0.0000000019
H,-1.4591859903,1.0410767168,-0.0000000006
H,0.7169877879,1.2135819345,1.2600852808
C,0.0910114679,-1.6653531039,0.7555908388
C,0.0910114679,-1.6653531047,-0.7555908369
H,1.0334859799,-1.5093733062,1.2575646639
H,-0.7713832429,-1.2567277813,1.2604254701
H,-0.7713832428,-1.2567277827,-1.2604254686
H,1.0334859799,-1.5093733076,-1.2575646621
H,0.716987788,1.2135819331,-1.2600852822
C,-0.092893287,-2.9522224367,0.0000000017
H,-1.0766384312,-3.3948727641,0.0000000019

H,0.7275738884,-3.6526755087,0.0000000021

geh3f_ch2o MP2= -2291.46688710 NIMAG= 0
Ge,0.7603785648,0.0558246026,0.
F,2.4101653884,0.6468920246,0.
H,0.6456218437,-0.7491979013,1.2700227245
H,-0.0670556509,1.322811602,0.
H,0.6456218437,-0.7491979013,-1.2700227245
O,-1.8222080092,-0.7286628289,0.
C,-2.6791102167,0.1340975361,0.
H,-3.745501192,-0.1235884364,0.
H,-2.4144859846,1.2009398305,0.

geh3f_co MP2= -2290.28797108 NIMAG= 0
Ge,0.,0.0893998758,0.0629463291
F,0.,1.5141066307,1.069650384
H,1.2593443404,0.1758509622,-0.7656787905
H,-1.2593443404,0.1758509622,-0.7656787905
H,0.,-1.0829091332,1.0146859992
C,0.,-2.4623095338,-1.740855082
O,0.,-3.3909588837,-2.3978373555

geh3f_h2cch2 MP2= -2255.55226629 NIMAG= 0
Ge,0.011537548,0.8751579377,0.
F,-0.0146792253,2.6218025113,0.
H,-1.4395781548,0.455026771,0.
H,0.7463683127,0.478615579,1.2587832562
C,-0.012873848,-2.3243729629,0.6676714363
C,-0.012873848,-2.3243729629,-0.6676714363
H,-0.9265236861,-2.1835348294,1.2289645638
H,0.8985035068,-2.47293326,1.2298580188
H,0.8985035068,-2.47293326,-1.2298580188
H,-0.9265236861,-2.1835348294,-1.2289645638
H,0.7463683127,0.478615579,-1.2587832562

geh3f_hcch MP2= -2254.31067140 NIMAG= 0
Ge,0.0113935618,0.777114993,0.
F,0.0320222374,2.5224934878,0.
H,0.7296977938,0.3621848384,1.2614846634
H,0.7296977938,0.3621848384,-1.2614846634
H,-1.4478949183,0.386593498,0.
C,0.5393718909,-2.4822956626,0.
C,-0.6739188391,-2.4758406819,0.
H,1.6018766654,-2.4993801533,0.
H,-1.7364411669,-2.476354751,0.

geh3f_n2 MP2= -2286.50924180 NIMAG= 0
Ge,0.,0.0812421121,0.0571694342

F,0.,1.5038874053,1.0624890547
H,1.2576348081,0.1608111061,-0.7751665275
H,-1.2576348081,0.1608111061,-0.7751665275
H,0.,-1.0962529057,1.0028625683
N,0.,-2.4410643664,-1.7257950154
N,0.,-3.3504842068,-2.3692087676

geh3f_nch MP2= -2270.40941224 NIMAG= 0
Ge,0.,0.0000000014,0.7941979287
F,0.,0.0000000014,2.5473085452
H,-0.0000000039,1.4604076099,0.4143523795
H,1.2647500907,-0.7302037995,0.4143523795
H,-1.2647500869,-0.7302038062,0.4143523795
N,0.,0.0000000014,-2.0494320626
C,0.,0.0000000014,-3.2149691133
H,0.,0.0000000014,-4.2807041098

geh3f_nh3 MP2= -2233.61360648 NIMAG= 0
Ge,0.,0.0000000015,0.5171902362
F,0.,0.0000000015,2.2823669301
H,1.2780312955,0.737871714,0.1988886954
H,-1.2780312955,0.737871714,0.1988886954
H,0.,-1.4757434235,0.1988886954
N,0.,0.0000000015,-2.1239391262
H,-0.8132149831,-0.4695098879,-2.5051494986
H,0.8132149831,-0.4695098879,-2.5051494986
H,0.,0.9390197803,-2.5051494986

geh3f_oh2 MP2= -2253.47795705 NIMAG= 0
Ge,0.,-0.0191653161,0.6094723974
F,0.,0.1046582924,2.3571756073
H,1.2670194174,0.6794406923,0.182416934
H,-1.2670194174,0.6794406923,0.182416934
H,0.,-1.5045381359,0.3416236359
O,0.,-0.0925269944,-2.1668619018
H,-0.7617020619,-0.394046035,-2.6713638688
H,0.7617020619,-0.394046035,-2.6713638688

geh3f_ph3 MP2= -2519.80807857 NIMAG= 0
Ge,0.,0.0000000015,1.0065211065
F,0.,0.0000000015,2.7549442815
H,1.2612261699,0.7281692702,0.6062971819
H,-1.2612261699,0.7281692702,0.6062971819
H,0.,-1.4563385359,0.6062971819
P,0.,0.0000000015,-2.395750295
H,-1.0362191562,-0.5982614073,-3.1427665448
H,1.0362191562,-0.5982614073,-3.1427665448
H,0.,1.1965228191,-3.1427665448

geh3f_sh2 MP2= -2576.05546460 NIMAG= 0
Ge,0.,0.0231499155,0.7714962507
F,0.,-0.0584504285,2.5168417391
H,1.2613027683,0.7678142394,0.4055267432
H,-1.2613027683,0.7678142394,0.4055267432
H,0.,-1.4140542113,0.3070236889
S,0.,0.2475616582,-2.5909865094
H,-0.9643376425,-0.6489510853,-2.8232988126
H,0.9643376425,-0.6489510853,-2.8232988126

TB complexes with SiH_3F as LA.
sih3f_c3h6 MP2= -508.25240334 NIMAG= 0
Si,-0.0361353126,1.6524462968,-0.0000000009
F,-0.1462069613,3.267044636,-0.0000000019
H,-1.4072232992,1.1117179669,-0.0000000006
H,0.6931502628,1.2558825093,1.2167368779
C,0.0778436605,-1.6883059549,0.7550796947
C,0.0778436605,-1.6883059558,-0.7550796928
H,1.0172298272,-1.5133232646,1.2565031752
H,-0.7920206353,-1.2950951029,1.2591121676
H,-0.7920206353,-1.2950951043,-1.2591121661
H,1.0172298272,-1.5133232661,-1.2565031735
H,0.6931502628,1.2558825079,-1.2167368793
C,-0.0804363586,-2.9794878097,0.0000000017
H,-1.0553679109,-3.4414780955,0.000000002
H,0.753301927,-3.6641817867,0.0000000021

sih3f_ch2o MP2= -504.94630185 NIMAG= 0
Si,0.7757359247,0.0729124489,0.
F,2.3184432126,0.5883140628,0.
H,0.6101603449,-0.7172051776,1.2293480425
H,-0.0409644222,1.3030217191,0.
H,0.6101603449,-0.7172051776,-1.2293480425
O,-1.7681639677,-0.7049440854,0.
C,-2.6491414517,0.1333287084,0.
H,-3.7076942698,-0.1551874769,0.
H,-2.4151091257,1.2068835082,0.

sih3f_co MP2= -503.76828401 NIMAG= 0
Si,0.,0.1267064987,0.0893991518
F,0.,1.4488421896,1.0238225211
H,1.2166268801,0.170052336,-0.7399402956
H,-1.2166268801,0.170052336,-0.7399402956
H,0.,-1.0461876464,0.9805705217
C,0.,-2.4605535379,-1.739643169
O,0.,-3.3893497412,-2.3967493265

sih3f_h2cch2 MP2= -469.03196992 NIMAG= 0
Si,0.0098802206,0.9218034555,0.

F,-0.0052953753,2.5416428209,0.
H,-1.3923295407,0.4693914701,0.
H,0.7162326776,0.4863733348,1.2174374018
C,-0.0107813932,-2.323948024,0.6675382662
C,-0.0107813932,-2.323948024,-0.6675382662
H,-0.9245502547,-2.18740646,1.2295078146
H,0.901822771,-2.4661924221,1.2293617811
H,0.901822771,-2.4661924221,-1.2293617811
H,-0.9245502547,-2.18740646,-1.2295078146
H,0.7162326776,0.4863733348,-1.2174374018

sih3f_hcch MP2= -467.79070145 NIMAG= 0
Si,0.0148516037,0.8255223893,0.
F,0.0347048343,2.4447421714,0.
H,0.7101263331,0.3730692464,1.2170776287
H,0.7101263331,0.3730692464,-1.2170776287
H,-1.3962839031,0.4006370712,0.
C,0.5346320759,-2.4789574564,0.
C,-0.6785560354,-2.4829931205,0.
H,1.5970693675,-2.4851456132,0.
H,-1.7408655935,-2.493243529,0.

sih3f_n2 MP2= -499.98974720 NIMAG= 0
Si,0.,0.1284760906,0.0906736094
F,0.,1.4492274245,1.0242369251
H,1.2143535824,0.1642655211,-0.7426216987
H,-1.2143535824,0.1642655211,-0.7426216987
H,0.,-1.0497818916,0.9749938172
N,0.,-2.4637632913,-1.7418819112
N,0.,-3.3731859361,-2.3853003854

sih3f_nch MP2= -483.88862293 NIMAG= 0
Si,0.,0.0000000014,0.8254514705
F,0.,0.0000000014,2.4516211396
H,-0.0000000037,1.4119811435,0.4102127108
H,1.2228115406,-0.7059905664,0.4102127108
H,-1.2228115368,-0.7059905729,0.4102127108
N,0.,0.0000000014,-2.0231596961
C,0.,0.0000000014,-3.1889122073
H,0.,0.0000000014,-4.2544649135

sih3f_nh3 MP2= -447.09355679 NIMAG= 0
Si,0.,0.0000000015,0.4680963781
F,0.,0.0000000015,2.1085883288
H,1.2446253013,0.7185847543,0.1431998064
H,-1.2446253013,0.7185847543,0.1431998064
H,0.,-1.437169504,0.1431998064
N,0.,0.0000000015,-2.0296904945
H,-0.8146865306,-0.4703594862,-2.4065030104

H,0.8146865306,-0.4703594862,-2.4065030104
H,0.,0.940718977,-2.4065030104

sih3f_oh2 MP2= -466.95760279 NIMAG= 0
Si,0.,-0.0115136461,0.617780887
F,0.,0.0718941723,2.2415553601
H,1.2272447307,0.6671062318,0.1725241025
H,-1.2272447307,0.6671062318,0.1725241025
H,0.,-1.4465126624,0.2854958202
O,0.,-0.0361064839,-2.1479968462
H,-0.7611308336,-0.4263986708,-2.5889367736
H,0.7611308336,-0.4263986708,-2.5889367736

sih3f_ph3 MP2= -733.28828257 NIMAG= 0
Si,0.,0.0000000015,1.0345746028
F,0.,0.0000000015,2.6558995871
H,1.2188502369,0.7037035139,0.5989710161
H,-1.2188502369,0.7037035139,0.5989710161
H,0.,-1.4074070233,0.5989710161
P,0.,0.0000000015,-2.3714885643
H,-1.0359504164,-0.5981062503,-3.1196047086
H,1.0359504164,-0.5981062503,-3.1196047086
H,0.,1.1962125051,-3.1196047086

sih3f_sh2 MP2= -789.53575453 NIMAG= 0
Si,0.,0.0209150199,0.8074244434
F,0.,-0.0538040551,2.4267556341
H,1.2193590736,0.7420046295,0.4042257783
H,-1.2193590736,0.7420046295,0.4042257783
H,0.,-1.3657581225,0.3094541571
S,0.,0.252557669,-2.5646909511
H,-0.9641263127,-0.6406633687,-2.8095762371
H,0.9641263127,-0.6406633687,-2.8095762371

TB complexes with F_2CO as LA.

f2co_c3h6 MP2= -430.26845727 NIMAG= 0
C,-0.1593420416,1.4828353441,0.
O,-1.3373602593,1.4516503774,0.
F,0.6169681663,1.5185416405,1.0619627684
F,0.6169681663,1.5185416405,-1.0619627684
C,0.0921194293,-1.5646296277,-0.7546665615
C,0.0921194293,-1.5646296277,0.7546665615
H,1.0367653721,-1.424437526,-1.2574061525
H,1.0367653721,-1.424437526,1.2574061525
H,-0.7641949451,-1.1392794215,-1.2558529787
H,-0.7641949451,-1.1392794215,1.2558529787
C,-0.1119538105,-2.8493840404,0.
H,0.6966319401,-3.5636255996,0.
H,-1.1027435121,-3.2762313052,0.

f2co_ch2o MP2= -426.96236883 NIMAG= 0
C,-0.390651735,-0.9215734113,0.
F,-1.1498634879,-1.0620671899,1.0626497009
F,-1.1498634879,-1.0620671899,-1.0626497009
O,0.7805701277,-0.7687561357,0.
O,-0.9604952236,1.606717258,0.
C,0.0832077074,2.2301750749,0.
H,0.0899952313,3.3282899127,0.
H,1.0538115577,1.7155804833,0.

f2co_co MP2= -425.78347973 NIMAG= 0
C,-0.0525356879,0.0909944807,-0.0837864915
F,1.2555208165,-0.0509098133,-0.053011559
F,-0.5836712167,-1.1127678288,-0.053011559
O,-0.6406277213,1.109599762,-0.1496265958
C,0.031327628,-0.0542610434,2.9388520969
O,-0.0032402329,0.005612248,4.0746315883

f2co_h2cch2 MP2= -391.04694286 NIMAG= 0
C,-1.0428088159,0.1121741166,0.
F,-0.9760727567,-0.6619096914,1.0621627297
F,-0.9760727567,-0.6619096914,-1.0621627297
O,-1.1674062054,1.2839944724,0.
C,2.0088770293,0.7277731479,0.
C,2.1370807438,-0.6005273746,0.
H,1.9534167152,1.2877871584,0.9232262884
H,2.1927782163,-1.1595927563,-0.9237944992
H,2.1927782163,-1.1595927563,0.9237944992
H,1.9534167152,1.2877871584,-0.9232262884

f2co_hcch MP2= -389.80626588 NIMAG= 0
C,-0.8492952012,0.1942269236,0.
F,-0.8637554861,-0.5822538917,1.0616879312
F,-0.8637554861,-0.5822538917,-1.0616879312
O,-0.8476387981,1.372842607,0.
C,2.2674996268,-0.6776842002,0.
C,2.2287676492,0.5346166146,0.
H,2.308188401,-1.7392112327,0.
H,2.1864607241,1.5963672136,0.

f2co_n2 MP2= -422.00523951 NIMAG= 0
C,-0.0528655688,0.0915658511,-0.0275503514
F,1.2550890868,-0.0502313308,0.0107489097
F,-0.5840429349,-1.1120546985,0.0107489097
O,-0.6408186001,1.1099303738,-0.096601128
N,0.0337844318,-0.0585163524,2.897875741
N,-0.0042496502,0.0073606101,4.0091960545

f2co_nch MP2= -405.90359544 NIMAG= 0
C,-0.0664160252,0.1135964836,-0.1030474269
F,1.2420047285,-0.0250379248,-0.1067260693
F,-0.5953261953,-1.0911598772,-0.1064711922
O,-0.6575242595,1.1322970041,-0.1405118221
N,0.0722089413,-0.1244927185,2.6517979347
C,0.006879583,-0.0115957575,3.8104127905
H,-0.0536774615,0.0930640875,4.8689326703

f2co_nh3 MP2= -369.10651329 NIMAG= 0
C,-0.0661265863,0.1145346072,-0.39594596
F,1.2425238894,-0.0256058572,-0.4328927451
F,-0.5990866218,-1.0888601816,-0.4328927451
O,-0.6555130177,1.1353818517,-0.4255535914
N,0.0384164316,-0.0665392113,2.2714390766
H,0.4781258359,0.7956105752,2.5707468249
H,0.4700446679,-0.8141412466,2.8008601306
H,-0.9280818876,-0.0162638325,2.5707468249

f2co_oh2 MP2= -388.97330426 NIMAG= 0
C,0.6385899299,0.1825062891,-0.0360280391
F,0.7067991424,-0.3599099806,1.1608065107
F,0.7325687611,-0.7842614046,-0.9197817528
O,0.5610872595,1.3356402695,-0.2708019751
O,-1.9941299895,-0.1213780858,-0.0521387079
H,-2.6584829147,-0.5634853413,0.4842345586
H,-2.2799831887,0.7968852537,-0.0862905944

f2co_ph3 MP2= -655.30278523 NIMAG= 0
C,-0.0788775533,0.1366199299,-0.5046768464
F,1.2281511962,-0.0031873256,-0.5624840904
F,-0.6113152932,-1.0652037984,-0.5624840904
O,-0.6673015766,1.1558002347,-0.4408256967
P,0.0592241267,-0.1025791966,2.8357024784
H,0.5850423328,1.0552465912,3.4472795155
H,0.566044455,-0.9804177553,3.8175159274
H,-1.2063915216,0.0209617731,3.4472795155

f2co_sh2 MP2= -711.55087161 NIMAG= 0
C,-0.0841187046,1.0115321766,0.0739841813
F,1.1047772418,1.0266463998,-0.4883809945
F,-0.9742251671,1.2484547491,-0.8649289244
O,-0.3091934444,0.8467964006,1.2195864975
S,-0.3053217948,-2.1852685022,-0.5885705525
H,-1.4147508625,-2.0940412769,0.1520714062
H,0.4684906716,-2.2953206069,0.4962383864

TB complexes with CO_2 as LA.

co2_c3h6 MP2= -305.95112535 NIMAG= 0
C,0.,0.,-2.7651179041
O,0.,1.1702754328,-2.773743135
O,0.,-1.1702754328,-2.773743135
C,-0.7536053461,0.,0.336247211
C,0.,0.,1.6385362497
C,0.7536053461,0.,0.336247211
H,-1.2534503975,0.912351711,0.0480397714
H,-1.2534503975,-0.912351711,0.0480397714
H,0.,-0.9108780886,2.216766497
H,0.,0.9108780886,2.216766497
H,1.2534503975,-0.912351711,0.0480397714
H,1.2534503975,0.912351711,0.0480397714

co2_ch2o MP2= -302.64293209 NIMAG= 0
C,1.5325774383,-0.041696476,0.
O,1.1938118565,-1.1636808451,0.
O,1.9041741789,1.0658915801,0.
O,-1.225950705,0.6223836553,0.
C,-1.8831719256,-0.399171617,0.
H,-2.9821888198,-0.3732829067,0.
H,-1.4038843733,-1.3882685306,0.

co2_co MP2= -301.46632975 NIMAG= 0
C
X,1,1.
O,1,r1,2,a1
O,1,r1,2,a1,3,180.,0
C,1,r2,3,a1,2,0.,0
O,1,r3,3,a1,2,0.,0

r1=1.17010856
r2=3.17978762
r3=4.3181958
a1=90.33062564

co2_h2cch2 MP2= -266.72961541 NIMAG= 0
C
X,1,1.
O,1,r1,2,a1
O,1,r1,2,a1,3,180.,0
X,1,r2,3,a1,2,0.,0
C,5,r3,1,90.,3,0.,0
C,5,r3,1,90.,4,0.,0
H,6,r4,5,a4,1,90.,0
H,6,r4,5,a4,1,-90.,0
H,7,r4,5,a4,1,90.,0
H,7,r4,5,a4,1,-90.,0

r1=1.17021235
r2=3.23554151
a1=90.31672357
r3=0.66708035
r4=1.08114688
a4=121.38575454

co2_hcch MP2= -265.48959829 NIMAG= 0
C
X,1,1.
O,1,r1,2,a1
O,1,r1,2,a1,3,180.,0
X,1,r2,3,a1,2,0.,0
C,5,r3,2,90.,3,0.,0
C,5,r3,2,90.,4,0.,0
H,5,r4,2,a4,3,0.,0
H,5,r4,2,a4,4,0.,0

r1=1.17005658
r2=3.16103704
a1=90.33572867
r3=0.60648046
r4=1.66867477
a4=89.86965387

co2_n2 MP2= -297.68834152 NIMAG= 0
C
X,1,1.
O,1,r1,2,a1
O,1,r1,2,a1,3,180.,0
N,1,r2,3,a1,2,0.,0
N,1,r3,3,a1,2,0.,0

r1=1.17012363
r2=3.08861797
r3=4.20269353
a1=90.16986213

co2_nch MP2= -281.58521726 NIMAG= 0
C
X,1,1.
O,1,r1,2,a1
O,1,r1,2,a1,3,180.,0
N,1,r2,3,a1,2,0.,0
C,1,r3,3,a1,2,0.,0
H,1,r4,3,a1,2,0.,0

r1=1.17000742
r2=2.94574106
r3=4.1123261

r4=5.17737059
a1=90.72938006

co2_nh3 MP2= -244.78682567 NIMAG= 0
C,0.,0.0004665877,-0.2666940594
O,1.169901849,0.0005830321,-0.2916575784
O,-1.169901849,0.0005830321,-0.2916575784
N,0.,-0.001269438,2.6704844026
H,0.,0.9373082293,3.0506929534
H,0.8132511066,-0.4679900242,3.0527681343
H,-0.8132511066,-0.4679900242,3.0527681343

co2_oh2 MP2= -264.65512043 NIMAG= 0
C,0.,0.0162053316,-0.2460927786
O,1.1697559497,0.0181200161,-0.2635269579
O,-1.1697559497,0.0181200161,-0.2635269579
O,0.,-0.2864916857,2.5100624061
H,0.7608465776,-0.3507852564,3.0945478716
H,-0.7608465776,-0.3507852564,3.0945478716

co2_ph3 MP2= -530.98557136 NIMAG= 0
C,1.5543921752,-0.047210421,0.
O,1.1611057891,-1.1504389706,0.
O,1.9579492192,1.050269129,0.
P,-1.889402341,0.3620216265,0.
H,-3.1361431111,1.0244587967,0.
H,-2.250057784,-0.5320888459,1.0314121893
H,-2.250057784,-0.5320888459,-1.0314121893

co2_sh2 MP2= -587.23346667 NIMAG= 0
C,0.0581978555,-1.3382741103,0.
O,0.0601145277,-1.3474896448,1.1701669686
O,0.0601145277,-1.3474896448,-1.1701669686
S,-0.4202077488,2.0261585564,0.
H,0.4677159186,2.2847249216,0.9649233188
H,0.4677159186,2.2847249216,-0.9649233188

ZB complexes with AsH_2F as LA.
ash2f_c3h6 MP2= -2453.01012547 NIMAG= 0
As,-0.2678658981,1.3229165776,0.
F,-0.0353693382,3.0667882667,0.
H,0.7445903607,1.0539185536,-1.0802850223
H,0.7445903607,1.0539185536,1.0802850223
C,0.0824687045,-1.7654255583,0.7569105446
C,0.0824687045,-1.7654255583,-0.7569105446
H,-0.8099059235,-1.4256078915,1.2607553293
H,1.0108474366,-1.5382076829,1.2579399353
H,1.0108474366,-1.5382076829,-1.2579399353

H,-0.8099059235,-1.4256078915,-1.2607553293
C,-0.0004571536,-3.061555514,0.
H,0.8718004806,-3.6964321768,0.
H,-0.9474275904,-3.5782323321,0.

ash2f_ch2o MP2= -2449.70397449 NIMAG= 0
As,-0.7638663348,0.1031434641,0.
F,-2.5282936966,0.1951504408,0.
H,-0.6895882498,-0.9326150504,1.0847035354
H,-0.6895882498,-0.9326150504,-1.0847035354
O,1.7721530257,-0.6175579433,0.
C,2.5055457554,0.3541309529,0.
H,2.0962219497,1.3733926296,0.
H,3.5962430241,0.2396237245,0.

ash2f_co MP2= -2448.52529775 NIMAG= 0
As,0.8650872411,0.2360864272,0.
F,2.5923083281,-0.1032632802,0.
H,0.5278827879,-0.7558137982,1.0801256063
H,0.5278827879,-0.7558137982,-1.0801256063
C,-2.0199424413,-0.0431765925,0.
O,-3.15610192,0.0069589395,0.

ash2f_h2cch2 MP2= -2413.79112412 NIMAG= 0
As,-0.1625112637,1.355578193,0.
F,0.1995064998,3.0846484958,0.
H,0.8218408624,1.0110663668,1.0843534623
H,0.8218408624,1.0110663668,-1.0843534623
C,0.6797891311,-1.6066335002,0.
C,-0.6555234844,-1.5086812783,0.
H,1.2394477784,-1.651885452,0.9239085477
H,1.2394477784,-1.651885452,-0.9239085477
H,-1.2180615977,-1.486306063,-0.9231544699
H,-1.2180615977,-1.486306063,0.9231544699

ash2f_hcch MP2= -2412.54881871 NIMAG= 0
As,-0.1493610787,1.4092235587,0.
F,0.2303200565,3.130116644,0.
H,0.829810181,1.0524338002,1.0849152919
H,0.829810181,1.0524338002,-1.0849152919
C,0.6001471509,-1.6230326798,0.
C,-0.6133855337,-1.5688890359,0.
H,1.6615851043,-1.6781921506,0.
H,-1.676115255,-1.5559636168,0.

ash2f_n2 MP2= -2444.74561631 NIMAG= 0
As,0.865908862,0.2519515994,0.
F,2.5755038731,-0.1479664461,0.

H,0.4915923386,-0.7287011689,1.0787267271
H,0.4915923386,-0.7287011689,-1.0787267271
N,-2.0751276032,-0.0252327067,0.
N,-3.1892248498,-0.0310640222,0.

ash2f_nch MP2= -2428.64661128 NIMAG= 0
As,0.8404689434,0.2155463905,-0.0020644976
F,2.5822102935,-0.0917090567,-0.0095283945
H,0.5438978107,-0.7742523568,1.089197884
H,0.5321602222,-0.7903246209,-1.0753485716
N,-1.8752868932,-0.0899691917,0.010961608
C,-3.0394281411,-0.0378789606,-0.0011913084
H,-4.1041537949,0.0088586343,-0.0120267197

ash2f_nh3 MP2= -2391.85190463 NIMAG= 0
As,-0.185547929,1.3731454316,0.
F,0.4465772821,3.0420022891,0.
H,0.7461833958,0.922371502,1.0890278243
H,0.7461833958,0.922371502,-1.0890278243
N,-0.4533824102,-1.2092304053,0.
H,-0.9878770607,-1.4913567244,0.8132671545
H,-0.9878770607,-1.4913567244,-0.8132671545
H,0.3996887162,-1.7560968557,0.

ash2f_oh2 MP2= -2411.71494618 NIMAG= 0
As,-0.1451485223,1.4325089612,-0.1102338085
F,0.4065830539,3.1083784179,-0.0167560853
H,0.5536977671,1.0016196944,1.1469727416
H,0.9975988267,0.9790080984,-0.9756313154
O,-0.332276835,-1.2742269666,-0.0582686513
H,-1.1338630424,-1.5996051593,-0.4796781619
H,0.3539132233,-1.8965385259,-0.3189909202

ash2f_ph3 MP2= -2678.04206436 NIMAG= 0
As,-0.0762240388,1.7323216435,0.
F,0.47867444,3.4126690753,0.
H,0.8632955537,1.2790445413,1.0856177163
H,0.8632955537,1.2790445413,-1.0856177163
P,-0.4113784657,-1.3314352693,0.
H,-1.2299558975,-1.8123241031,1.0413619109
H,-1.2299558975,-1.8123241031,-1.0413619109
H,0.4665276378,-2.4348417712,0.

ash2f_sh2 MP2= -2734.29322517 NIMAG= 0
As,-0.1128749751,1.5597744279,0.
F,0.4442065409,3.235057628,0.
H,0.8318777549,1.1081464615,1.0804087347
H,0.8318777549,1.1081464615,-1.0804087347

S,-0.2663284963,-1.599519865,0.
H,-1.1929673762,-1.6337391924,0.9636231466
H,-1.1929673762,-1.6337391924,-0.9636231466

ZB complexes with PH_2F as LA.

ph2f_c3h6 MP2= -559.45746835 NIMAG= 0
P,-0.2548160772,1.3848641469,0.
F,-0.0729336984,3.0011421671,0.
H,0.6788023606,1.0920579346,-1.0223365544
H,0.6788023606,1.0920579346,1.0223365544
C,0.1210611529,-1.7754205305,0.7558293921
C,0.1210611529,-1.7754205305,-0.7558293921
H,-0.7533876634,-1.3892930862,1.2574259601
H,1.0593756058,-1.594354614,1.2576194772
H,1.0593756058,-1.594354614,-1.2576194772
H,-0.7533876634,-1.3892930862,-1.2574259601
C,-0.0255705613,-3.0671879174,0.
H,0.8145938079,-3.7440825738,0.
H,-0.9962947177,-3.5378755717,0.

ph2f_ch2o MP2= -556.15190534 NIMAG= 0
P,-0.7793534689,0.073388483,0.
F,-2.4081379467,0.1941635108,0.
H,-0.6710407959,-0.8905872688,1.0260413392
H,-0.6710407959,-0.8905872688,-1.0260413392
O,1.7641146532,-0.6507263069,0.
C,2.4696951798,0.3407414206,0.
H,2.0320731128,1.348107798,0.
H,3.5639715758,0.257386413,0.

ph2f_co MP2= -554.97369570 NIMAG= 0
P,0.8819907536,0.1732122646,0.
F,2.4922496769,-0.0610197052,0.
H,0.5563044027,-0.7485647983,1.022490224
H,0.5563044027,-0.7485647983,-1.022490224
C,-2.0145331627,-0.1065567741,0.
O,-3.137176841,0.0780123546,0.

ph2f_h2cch2 MP2= -520.23860137 NIMAG= 0
P,-0.1257369058,1.3858649347,0.
F,0.1784618806,2.9885241405,0.
H,0.7788765508,1.0237024729,1.0254420856
H,0.7788765508,1.0237024729,-1.0254420856
C,0.6895612847,-1.6195762602,0.
C,-0.6415647163,-1.4842573874,0.
H,1.24820266,-1.6815395286,0.9237261749
H,1.24820266,-1.6815395286,-0.9237261749
H,-1.2035824978,-1.4421098511,-0.9225724532
H,-1.2035824978,-1.4421098511,0.9225724532

ph2f_hcch MP2= -518.99700059 NIMAG= 0
P,-0.1170140946,1.4401179618,0.
F,0.2041974329,3.0371178906,0.
H,0.7816628209,1.0668797325,1.0260441103
H,0.7816628209,1.0668797325,-1.0260441103
C,0.6211606162,-1.6424482529,0.
C,-0.5887586201,-1.5376629849,0.
H,1.6791518929,-1.7420482034,0.
H,-1.6492832482,-1.4706605463,0.

ph2f_n2 MP2= -551.19444914 NIMAG= 0
P,0.8935634528,0.1881181142,0.
F,2.4911366729,-0.1048711015,0.
H,0.532372225,-0.7230012317,1.021013081
H,0.532372225,-0.7230012317,-1.021013081
N,-2.0916122537,-0.0953428608,0.
N,-3.196424614,0.0484634411,0.

ph2f_nch MP2= -535.09382393 NIMAG= 0
P,0.8705715167,0.1639224856,-0.004685247
F,2.4908343638,-0.0528629762,-0.0033188464
H,0.5674384532,-0.7509496953,1.0285219405
H,0.5662658381,-0.772299975,-1.0182510261
N,-1.8813400027,-0.1892854279,0.0004702974
C,-3.0382961057,-0.0455246126,-0.0007792708
H,-4.0956056228,0.0872710395,-0.0019578476

ph2f_nh3 MP2= -498.29816443 NIMAG= 0
P,-0.1305483849,1.38828543,0.
F,0.3961007039,2.9491835213,0.
H,0.7250138243,0.9395396185,1.0292171371
H,0.7250138243,0.9395396185,-1.0292171371
N,-0.4186098281,-1.2038565957,0.
H,-0.9771348741,-1.4361550233,0.8126537603
H,-0.9771348741,-1.4361550233,-0.8126537603
H,0.380998623,-1.8263025633,0.

ph2f_oh2 MP2= -518.16264153 NIMAG= 0
P,-0.0871640968,1.4438207282,-0.1179635375
F,0.3522428424,3.0147730585,-0.0393826858
H,0.5027795648,1.0306771923,1.095882158
H,1.0060676348,0.9938485953,-0.8920838341
O,-0.2654746526,-1.2991105092,-0.026190551
H,-1.1274299133,-1.4776705302,-0.4151178831
H,0.3194830921,-1.9551940148,-0.4177298675

ph2f_ph3 MP2= -784.49212681 NIMAG= 0
P,-0.0212302546,1.7076261988,0.
F,0.3597394857,3.2962858705,0.
H,0.8647515755,1.3046650532,1.0261059831
H,0.8647515755,1.3046650532,-1.0261059831
P,-0.3238342258,-1.337215878,0.
H,-1.2029088026,-1.7022929366,1.0396355591
H,-1.2029088026,-1.7022929366,-1.0396355591
H,0.3854518711,-2.5573011564,0.

ph2f_sh2 MP2= -840.74143109 NIMAG= 0
P,-0.0644229866,1.566644295,0.
F,0.4099835739,3.1275868121,0.
H,0.8014317201,1.1124590025,1.0222570373
H,0.8014317201,1.1124590025,-1.0222570373
S,-0.2513380026,-1.6235420841,0.
H,-1.1775401684,-1.5745014824,0.9629402453
H,-1.1775401684,-1.5745014824,-0.9629402453

ZB complexes with NO_2F as LA.

no2f_c3h6 MP2= -422.14141189 NIMAG= 0
N,0.0872307196,1.4996090642,0.
F,-1.4352937616,1.3716789097,0.
O,0.5040591765,1.5387233536,1.1003491585
O,0.5040591765,1.5387233536,-1.1003491585
C,0.1249977751,-1.5696529755,-0.7547518267
C,0.1249977751,-1.5696529755,0.7547518267
H,1.0745962314,-1.4683280803,-1.2583301078
H,1.0745962314,-1.4683280803,1.2583301078
H,-0.7131182125,-1.1074825401,-1.2536570502
H,-0.7131182125,-1.1074825401,1.2536570502
C,-0.1304997217,-2.845191908,0.
H,0.6484457421,-3.5917030529,0.
H,-1.137668067,-3.2318283354,0.

no2f_ch2o MP2= -418.83469117 NIMAG= 0
N,-0.6629963211,-1.0178983882,0.
O,-1.0617072027,-1.0941111516,1.1010450892
O,-1.0617072027,-1.0941111516,-1.1010450892
F,0.8741230904,-0.7559272275,0.
O,-0.9783517023,1.6748997597,0.
C,0.084430569,2.266108783,0.
H,0.1247418013,3.3644333098,0.
H,1.0381776584,1.7229048681,0.

no2f_co MP2= -417.65634258 NIMAG= 0
N,0.0859524801,-0.1488740627,-0.0820091075
O,1.2478187631,0.038617841,-0.1076221912
O,-0.6573534134,-1.0613338274,-0.1076221912
F,-0.6746715114,1.1685653366,0.0109117793

C,0.0725814913,-0.1257148307,2.9261403293
O,-0.0672903052,0.1165502275,4.0291820945

no2f_h2cch2 MP2= -382.92046705 NIMAG= 0
N,-0.2330574418,1.0127795496,0.
O,-0.6495697701,0.981015297,1.1001982842
O,-0.6495697701,0.981015297,-1.1001982842
F,1.2926748871,1.1115166585,0.
C,0.0645814452,-2.0473544266,0.6675290308
C,0.0645814452,-2.0473544266,-0.6675290308
H,-0.7675330687,-2.4479174128,1.2299514489
H,0.9006397742,-1.6505217313,-1.2267649926
H,-0.7675330687,-2.4479174128,-1.2299514489
H,0.9006397742,-1.6505217313,1.2267649926

no2f_hcch MP2= -381.67946587 NIMAG= 0
N,-0.8786348475,-0.0010639134,0.
O,-0.9034481132,-0.413113581,1.1008439521
O,-0.9034481132,-0.413113581,-1.1008439521
F,-0.7870525022,1.538214665,0.
C,2.3800951243,-0.7432596525,0.
C,2.1432418164,0.4466084375,0.
H,2.6018013246,-1.7821155151,0.
H,1.9139167527,1.4844932622,0.

no2f_n2 MP2= -413.87808835 NIMAG= 0
N,0.0860359497,-0.1490186361,-0.0223232268
O,1.2484139,0.0376878117,-0.0495971157
O,-0.6568455519,-1.0623142463,-0.0495971157
F,-0.6732448466,1.1660942798,0.0782040013
N,0.0761216316,-0.1318465335,2.8850452683
N,-0.0734034619,0.1271385255,3.9580985993

no2f_nch MP2= -397.77605238 NIMAG= 0
N,1.04101942,-0.21305966,0.
O,1.02636966,-0.62931995,1.09982357
O,1.02636966,-0.62931995,-1.09982357
F,1.11048426,1.316145,0.
N,-1.77618806,-0.21024291,0.
C,-2.89301871,0.12540965,0.
H,-3.91224966,0.43528259,0.

no2f_nh3 MP2= -360.97858252 NIMAG= 0
N,0.7280815448,-0.0583964574,0.
O,0.8357949909,-0.4509961337,1.1005301914
O,0.8357949909,-0.4509961337,-1.1005301914
F,0.3478255899,1.4495800086,0.
N,-2.0697170579,-0.4288597868,0.

H,-2.6109516543,-0.6997238365,-0.8117967763
H,-2.0307298802,0.5839869258,0.
H,-2.6109516543,-0.6997238365,0.8117967763

no2f_oh2 MP2= -380.84651544 NIMAG= 0
N,0.6779736296,-0.0885960014,0.0114011255
O,0.8392188156,-0.2843636291,1.1557578685
O,0.7602093792,-0.6426680069,-1.0176431294
F,0.2180865767,1.4042754945,-0.2178431758
O,-2.0572477754,-0.2685759269,-0.0265090265
H,-2.8664327693,-0.3061169621,0.4905401688
H,-1.8653588564,0.6720420318,-0.115703831

no2f_ph3 MP2= -647.17596120 NIMAG= 0
N,0.0467079738,-0.0809005838,-0.4872657232
O,1.197251753,0.1276094819,-0.6138578174
O,-0.7091389298,-0.9730456917,-0.6138578174
F,-0.6849890616,1.1864358575,-0.0168870169
P,0.1482470914,-0.2567714945,2.8162342304
H,0.5961460347,1.0354110534,3.162808267
H,0.4751165992,-0.8229260896,4.0673243327
H,-1.194765293,0.0014279165,3.162808267

no2f_sh2 MP2= -703.42411676 NIMAG= 0
N,1.2054456973,0.2090252035,-0.0186052865
O,1.2343836491,0.7618384176,-1.055019133
O,1.233030447,0.4683527976,1.1273075485
F,1.1023789691,-1.320757,-0.2243161352
S,-2.0944321773,0.0338982024,-0.1131498077
H,-2.231672116,-0.2304307554,1.1900632921
H,-1.4585934692,-1.1223488656,-0.3336914782

ZB complexes with N_2O as LA.
n2o_c3h6 MP2= -302.03690972 NIMAG= 0
N,0.,-0.0935308733,-2.7500535747
N,0.,1.0584116171,-2.8349903469
O,0.,-1.2710173214,-2.6616788002
C,-0.7530614623,0.0798577669,0.3308389137
C,0.,-0.0554624179,1.6266357848
C,0.7530614623,0.0798577669,0.3308389137
H,-1.2526459745,1.016858363,0.1371522244
H,-1.2535332997,-0.7980489052,-0.0495800039
H,0.,-1.0213780661,2.107400306
H,0.,0.7903794994,2.2964501873
H,1.2535332997,-0.7980489052,-0.0495800039
H,1.2526459745,1.016858363,0.1371522244

n2o_ch2o MP2= -298.72812439 NIMAG= 0

N,1.5544260465,-0.0634906453,0.
N,1.2403334545,-1.1758445964,0.
O,1.884334346,1.0676231061,0.
O,-1.249697685,0.63854316,0.
C,-1.8950129681,-0.3905038648,0.
H,-2.994590859,-0.3792069435,0.
H,-1.404424685,-1.3749453561,0.

n2o_co MP2= -297.55178578 NIMAG= 0
N,0.018216122,0.,0.0136978153
N,1.1717391505,0.,-0.0424977478
O,-1.1607333739,0.,0.0741656263
C,0.021258375,0.,3.1513776633
O,-0.0458278106,0.,4.2878410976

n2o_h2cch2 MP2= -262.81555357 NIMAG= 0
N,0.0183520245,0.,0.0413574516
N,1.1735245534,0.,0.0150983855
O,-1.1611060285,0.,0.0827257672
C,0.6595230081,0.,3.206561479
C,-0.6750375094,0.,3.2130809907
H,1.2225269494,0.9229903792,3.2045944268
H,1.2225269494,-0.9229903792,3.2045944268
H,-1.2378372177,-0.9231017937,3.216464404
H,-1.2378372177,0.9231017937,3.216464404

n2o_hcch MP2= -261.57548492 NIMAG= 0
N,0.0141308819,0.,0.0226004168
N,1.1690112317,0.,0.0000834708
O,-1.1653295032,0.,0.0589078614
C,0.5987552735,0.,3.1343938673
C,-0.6145916074,0.,3.1352017677
H,1.6609357625,0.,3.1341299005
H,-1.6766326253,0.,3.1371825714

n2o_n2 MP2= -293.77384868 NIMAG= 0
N,0.0205868586,0.,0.0211387556
N,1.1716803399,0.,-0.0741277508
O,-1.1558577254,0.,0.121714472
N,0.0546774669,0.,3.0558779891
N,-0.096985039,0.,4.1596343268

n2o_nch MP2= -277.67049150 NIMAG= 0
N,0.0020287062,0.,-0.0080080874
N,1.1546823418,0.,-0.0735370833
O,-1.1764131619,0.,0.0550862445
N,0.0258105695,0.,2.9447296323
C,-0.0171600031,0.,4.1106964129

H,-0.0566434569,0.,5.1752164502

n2o_nh3 MP2= -240.87159783 NIMAG= 0
N,0.0128450789,-0.0005095472,-0.3255146249
N,1.1519556832,-0.1728673756,-0.3927486661
O,-1.1524993024,0.1756488683,-0.2639579182
N,0.0193255249,0.0033173658,2.7035669344
H,-0.1557446383,0.9241090986,3.08680436
H,0.8624896307,-0.3393627473,3.1473720371
H,-0.7383719769,-0.5886442678,3.0211822861

n2o_oh2 MP2= -260.74004611 NIMAG= 0
N,0.91044361,0.09175932,0.
N,0.79046141,1.24020264,0.
O,1.03850561,-1.08033837,0.
O,-1.90772201,-0.09909312,0.
H,-2.27676685,0.78929564,0.
H,-2.67583708,-0.67757738,0.

n2o_ph3 MP2= -527.07112541 NIMAG= 0
N,1.5432833505,-0.0639651048,0.
N,1.1730882467,-1.1591143896,0.
O,1.9138114908,1.0558410032,0.
P,-1.8793813925,0.3706482962,0.
H,-3.1313795678,1.023630518,0.
H,-2.2358179478,-0.5260586289,1.030920265
H,-2.2358179478,-0.5260586289,-1.030920265

n2o_sh2 MP2= -583.31886252 NIMAG= 0
N,0.0590146008,-1.3457413878,0.0135928705
N,0.1085649481,-1.3551894885,1.1675826631
O,0.0083887911,-1.3321049404,-1.1655217435
S,-0.4160053687,2.0171290148,-0.0361214942
H,0.4195239423,2.2785931852,0.9737649374
H,0.5141640858,2.2996686163,-0.9532972333

YB complexes with SO_3 as LA.
so3_c3h6 MP2= -740.69544391 NIMAG= 0
S,-0.0928997636,1.1440854734,-0.0000000012
O,-1.5331421256,1.0262225255,-0.0000000011
O,0.6219025508,1.2526194665,1.2507987576
O,0.6219025508,1.2526194637,-1.2507987603
C,0.0842128818,-1.7472515949,-0.7615569092
C,0.0842128818,-1.7472515932,0.761556913
H,1.0126925257,-1.5179271554,-1.2611058368
H,1.0126925257,-1.5179271527,1.2611058401
H,-0.8088690945,-1.4123698194,-1.2676491046
H,-0.8088690945,-1.4123698167,1.2676491077
C,0.0031438731,-3.0389461209,0.0000000033

H,0.8770699351,-3.6714046424,0.000000004
H,-0.9438494627,-3.5555377846,0.0000000039

so3_ch2o MP2= -737.39374350 NIMAG= 0
S,-0.508903606,-0.7987674473,0.
O,-1.1955990802,-1.007742589,1.2505371656
O,-1.1955990802,-1.007742589,-1.2505371656
O,0.9307804666,-0.6370998089,0.
O,-0.8935806659,1.4433747009,0.
C,0.1074021662,2.1447193357,0.
H,0.0065733604,3.2333623848,0.
H,1.105637129,1.6961948126,0.

so3_co MP2= -736.20940201 NIMAG= 0
S
X,1,1.
O,1,r1,2,a1
O,1,r1,2,a1,3,120.,0
O,1,r1,2,a1,3,-120.,0
C,1,r2,3,a1,2,0.,0
O,1,r3,3,a1,2,0.,0

r1=1.44341722
a1=90.73545533
r2=2.80835426
r3=3.94478729

so3_h2cch2 MP2= -701.47590243 NIMAG= 0
S,0.0114675712,-0.0094164298,0.7621286443
O,1.1275705028,-0.9258884336,0.7543679763
O,-1.3380584772,-0.5188931132,0.8283407823
O,0.2486522058,1.4134435406,0.8283407822
C,0.4243402333,0.518090483,-2.0468743392
C,-0.4256331901,-0.517028785,-2.0468743391
H,1.4908697456,0.3670680193,-1.9483295724
H,-1.4916252675,-0.368360501,-2.1479782798
H,-0.0699980664,-1.5337965283,-1.9483295723
H,0.0711188654,1.534789078,-2.1479782799

so3_hcch MP2= -700.23341942 NIMAG= 0
S,-0.7377473776,0.0464828373,0.
O,-0.7916172304,-0.6743445247,1.2497808493
O,-0.7139976165,1.490525319,0.
O,-0.7916172304,-0.6743445247,-1.2497808493
C,2.1162662183,-0.6242121355,0.
C,2.1808394261,0.5886420053,0.
H,2.0696142999,-1.6866202971,0.
H,2.2347309406,1.6505214604,0.

so3_n2 MP2= -732.42940785 NIMAG= 0
S
X,1,1.
O,1,r1,2,a1
O,1,r1,2,a1,3,120.,0
O,1,r1,2,a1,3,-120.,0
N,1,r2,3,a1,2,0.,0
N,1,r3,3,a1,2,0.,0

r1=1.44406804
a1=90.26989728
r2=2.86355641
r3=3.97737856

so3_nch MP2= -716.33230056 NIMAG= 0
S
X,1,1.
O,1,r1,2,a1
O,1,r1,2,a1,3,120.,0
O,1,r1,2,a1,3,-120.,0
N,1,r2,3,a1,2,0.,0
C,1,r3,3,a1,2,0.,0
H,1,r4,3,a1,2,0.,0

r1=1.44264236
a1=91.68580725
r2=2.54676836
r3=3.71063399
r4=4.77653414

so3_nh3 MP2= -679.55401716 NIMAG= 0
S
X,1,1.
O,1,r1,2,a1
O,1,r1,2,a1,3,120.,0
O,1,r1,2,a1,3,-120.,0
N,1,r2,3,a1,2,0.,0
H,6,r3,1,a3,3,60.,0
H,6,r3,1,a3,4,60.,0
H,6,r3,1,a3,5,60.,0

r1=1.44697703
a1=96.92733347
r2=2.01740884
r3=1.01531837
a3=108.70183783

so3_oh2 MP2= -699.40337500 NIMAG= 0
S,-0.03199369,-0.45373963,0.

O,-0.75831273,-0.4111932,1.2476959
O,-0.75831273,-0.4111932,-1.2476959
O,1.38087424,-0.73379101,0.
O,0.25561338,1.90375216,0.
H,-0.22478252,2.23940385,0.76717288
H,-0.22478252,2.23940385,-0.76717288

so3_ph3 MP2= -965.73505822 NIMAG= 0
S
X,1,1.
O,1,r1,2,a1
O,1,r1,2,a1,3,120.,0
O,1,r1,2,a1,3,-120.,0
P,1,r2,3,a1,2,0.,0
H,6,r3,1,a3,3,60.,0
H,6,r3,1,a3,4,60.,0
H,6,r3,1,a3,5,60.,0

r1=1.44761915
a1=96.04690799
r2=2.50102526
r3=1.40193255
a3=117.12313792

so3_sh2 MP2= -1021.98101818 NIMAG= 0
S,-0.0091375056,0.8342960041,0.
O,0.7050205418,0.9455993178,-1.2504431708
O,-1.4553534467,0.8401709651,0.
O,0.7050205418,0.9455993178,1.2504431708
S,0.1285689588,-1.94625764,0.
H,-0.7942305751,-2.0303043124,-0.9663891361
H,-0.7942305751,-2.0303043124,0.9663891361

YB complexes with SeF_2 as LA.
sef2_c3h6 MP2= -2717.18130223 NIMAG= 0
Se,0.391689484,1.2547479243,0.
F,0.3616613853,2.9944480307,0.
F,-1.3358901714,1.0757746087,0.
C,0.1899564601,-1.698596318,0.7598876444
C,0.1899564601,-1.698596318,-0.7598876444
H,-0.6541384237,-1.2527742458,1.2634983633
H,1.1382735,-1.5887082083,1.2635986599
H,1.1382735,-1.5887082083,-1.2635986599
H,-0.6541384237,-1.2527742458,-1.2634983633
C,-0.049344696,-2.9724317982,0.
H,0.7397268211,-3.7080403015,0.
H,-1.0524127905,-3.3690982831,0.

sef2_ch2o MP2= -2713.87736099 NIMAG= 0

Se,-0.490857245,0.7259337981,0.
F,-0.1564195562,2.4431540431,0.
F,1.2022599701,0.2965067634,0.
O,-0.6830129663,-1.7733187951,0.
C,0.3009650784,-2.4932435697,0.
H,1.3113696944,-2.0714796919,0.
H,0.1901881546,-3.5834453281,0.

sef2_co MP2= -2712.69496907 NIMAG= 0
Se,0.4438130998,-0.7227618862,0.
F,0.3362889389,-2.458190853,0.
F,-1.2741256835,-0.474151089,0.
C,0.2639676456,2.1487000389,0.
O,0.0418663591,3.2637303794,0.

sef2_h2cch2 MP2= -2677.96405303 NIMAG= 0
Se,0.4122598107,0.2999599525,0.
F,0.1968880681,2.0505264904,0.
F,-1.3146244055,0.055416378,0.
C,0.319484228,-2.2644240084,0.6744429519
C,0.319484228,-2.2644240084,-0.6744429519
H,-0.6006016274,-2.1805981156,1.234930748
H,1.2355269762,-2.3940244901,1.2339281871
H,1.2355269762,-2.3940244901,-1.2339281871
H,-0.6006016274,-2.1805981156,-1.234930748

sef2_hcch MP2= -2676.72045984 NIMAG= 0
Se,0.3599114734,0.4415241693,0.
F,0.2869296611,2.1882732494,0.
F,-1.3668521544,0.259719557,0.
C,0.3187390458,-2.3321382715,0.6083075278
C,0.3187390458,-2.3321382715,-0.6083075278
H,0.3099650213,-2.3583576593,1.6712028247
H,0.3099650213,-2.3583576593,-1.6712028247

sef2_n2 MP2= -2708.91527521 NIMAG= 0
Se,0.4419662181,-0.7308738285,0.
F,0.3278709869,-2.4610447954,0.
F,-1.2684766577,-0.4504433413,0.
N,0.2699381733,2.2124078498,0.
N,0.0197169694,3.2979472554,0.

sef2_nch MP2= -2692.81726931 NIMAG= 0
Se,0.4415165769,-0.6472748281,0.
F,0.3055470864,-2.3891923479,0.
F,-1.2773231388,-0.3929568567,0.
N,0.337867502,2.000860851,0.
C,0.0825915871,3.1372704347,0.

H,-0.1539720838,4.1765041171,0.

sef2_nh3 MP2= -2656.02740916 NIMAG= 0
Se,0.4753348781,0.2149904757,0.
F,0.2146434124,1.9699700825,0.
F,-1.2563832484,-0.0574641401,0.
N,0.3100205192,-2.1667468538,0.
H,1.1772478461,-2.6909858303,0.
H,-0.2233345037,-2.4357229272,0.8183139638
H,-0.2233345037,-2.4357229272,-0.8183139638

sef2_oh2 MP2= -2675.88784495 NIMAG= 0
Se,0.403378484,0.2994827785,0.
F,0.2831932659,2.0432363631,0.
F,-1.3252900053,0.0734015797,0.
O,0.3151488983,-2.2494133165,0.
H,-0.1969303549,-2.5407832402,0.7628756672
H,-0.1969303549,-2.5407832402,-0.7628756672

sef2_ph3 MP2= -2942.21796721 NIMAG= 0
Se,0.4405721699,0.6814557474,0.
F,0.1106371346,2.4084013789,0.
F,-1.2626247078,0.2914467797,0.
P,0.4023877632,-2.2217555101,0.
H,1.4506299369,-3.1625847436,0.
H,-0.3336550506,-2.7993346249,1.0506279184
H,-0.3336550506,-2.7993346249,-1.0506279184

sef2_sh2 MP2= -2998.46518411 NIMAG= 0
Se,0.4443776136,0.4795863407,0.
F,0.1580400497,2.2058424588,0.
F,-1.2548245953,0.0940824211,0.
S,0.5919966923,-2.510769952,0.
H,-0.3312202308,-2.5756699991,0.9657535445
H,-0.3312202308,-2.5756699991,-0.9657535445

YB complexes with SF_2 as LA.
sf2_c3h6 MP2= -714.76977359 NIMAG= 0
S,0.3456569944,1.3414574906,0.
F,0.3882797769,2.9522225829,0.
F,-1.2551843108,1.1788499675,0.
C,0.1707929898,-1.7128723016,0.7566833262
C,0.1707929898,-1.7128723016,-0.7566833262
H,-0.6851230428,-1.2884210673,1.2589848362
H,1.1152008935,-1.5726907408,1.2600718274
H,1.1152008935,-1.5726907408,-1.2600718274
H,-0.6851230428,-1.2884210673,-1.2589848362
C,-0.03202941,-2.9962624846,0.

H,0.7776707604,-3.7092637863,0.
H,-1.0225223866,-3.4237929271,0.

sf2_ch2o MP2= -711.46396947 NIMAG= 0
S,-0.4182015314,0.8003969414,0.
F,-0.1562127443,2.3972258341,0.
F,1.1411463833,0.3697230496,0.
O,-0.6932204224,-1.8059762204,0.
C,0.2941218899,-2.5169919213,0.
H,1.3045547222,-2.0896041182,0.
H,0.2023048328,-3.6106663453,0.

sf2_co MP2= -710.28459459 NIMAG= 0
S,0.36373917,-0.82056331,0.
F,0.33739952,-2.43043013,0.
F,-1.22777669,-0.58972477,0.
C,0.25700953,2.23697566,0.
O,0.08143883,3.36106914,0.

sf2_h2cch2 MP2= -675.54985450 NIMAG= 0
S,0.2991652652,0.5391672876,0.
F,0.1828704494,2.1524580507,0.
F,-1.2864566747,0.250214506,0.
C,0.3350566483,-2.3662211965,0.6688106149
C,0.3350566483,-2.3662211965,-0.6688106149
H,-0.5838766144,-2.2688162921,1.2299978873
H,1.2527017589,-2.4719776464,1.2308036851
H,1.2527017589,-2.4719776464,-1.2308036851
H,-0.5838766144,-2.2688162921,-1.2299978873

sf2_hcch MP2= -674.30846507 NIMAG= 0
S,0.2755494241,0.5632499634,0.
F,0.27221103,2.1781797169,0.
F,-1.3209783582,0.3701393129,0.
C,0.3294267547,-2.3972932392,0.6070769061
C,0.3294267547,-2.3972932392,-0.6070769061
H,0.3258805432,-2.4042297257,1.669761232
H,0.3258805432,-2.4042297257,-1.669761232

sf2_n2 MP2= -706.50596616 NIMAG= 0
S,0.36002258,-0.79461956,0.
F,0.34064574,-2.40206678,0.
F,-1.22930233,-0.56012117,0.
N,0.23071768,2.25993183,0.
N,0.08893202,3.36486882,0.

sf2_nch MP2= -690.40507740 NIMAG= 0

S,0.35753864,-0.74223254,0.
F,0.33241239,-2.35928047,0.
F,-1.23748527,-0.52931986,0.
N,0.28214042,2.05528267,0.
C,0.08968666,3.20507668,0.
H,-0.08806531,4.25568489,0.

sf2_nh3 MP2= -653.61095868 NIMAG= 0
S,0.3825583315,0.2909110575,0.
F,0.2359973266,1.9224031429,0.
F,-1.2192587526,0.0345283077,0.
N,0.3078792357,-2.1899994705,0.
H,1.19144192,-2.6851743654,0.
H,-0.2122118306,-2.4871753963,0.8167185588
H,-0.2122118306,-2.4871753963,-0.8167185588

sf2_oh2 MP2= -673.47510757 NIMAG= 0
S,0.3147198601,0.3953991978,0.
F,0.2923741444,2.0127415586,0.
F,-1.286862615,0.1952504941,0.
O,0.3042913422,-2.2737170011,0.
H,-0.1709313702,-2.6226726143,0.761177332
H,-0.1709313702,-2.6226726143,-0.761177332

sf2_ph3 MP2= -939.80513733 NIMAG= 0
S,0.3159565161,0.8740653442,0.
F,0.1389616718,2.4812525621,0.
F,-1.2570776781,0.5198436796,0.
P,0.4114123875,-2.3209894466,0.
H,1.4714437502,-3.2515910942,0.
H,-0.3032511237,-2.9521315821,1.0388279225
H,-0.3032511237,-2.9521315821,-1.0388279225

sf2_sh2 MP2= -996.05315253 NIMAG= 0
S,0.3569063563,0.5749387583,0.
F,0.1696118378,2.1801935703,0.
F,-1.2138185388,0.2080412114,0.
S,0.6042978058,-2.5739958027,0.
H,-0.3199240812,-2.6358882335,0.9642803941
H,-0.3199240812,-2.6358882335,-0.9642803941

YB complexes with SO_2 as LA.

so2_c3h6 MP2= -665.59672138 NIMAG= 0
S,0.389252484,-1.4666115595,0.
O,-0.3447592699,-1.6210054512,1.2573107703
O,-0.3447592699,-1.6210054512,-1.2573107703
C,0.7231542492,1.6930142828,0.
C,-0.7775219254,1.5168085686,0.

H,1.2568042811,1.4732818792,-0.9128478393
H,-1.2374623479,1.1666760345,-0.9119877043
H,1.2568042811,1.4732818792,0.9128478393
H,-1.2374623479,1.1666760345,0.9119877043
C,-0.1833081877,2.8960131999,0.
H,-0.2480928484,3.4698152116,-0.9113919239
H,-0.2480928484,3.4698152116,0.9113919239

so2_ch2o MP2= -662.29043897 NIMAG= 0
S,-1.0179719444,-0.01849895,0.3465712172
O,-1.2902024584,1.3303146815,-0.1500196024
O,-0.7718052641,-1.0724032866,-0.6433643158
O,1.698665913,0.3018839733,0.5411767961
C,2.1758021812,-0.2235355894,-0.4469299132
H,1.5476637179,-0.7847220115,-1.1533026638
H,3.2495638548,-0.1521928172,-0.6625535181

so2_co MP2= -661.11032134 NIMAG= 0
S,0.4436160291,-1.1737192785,0.
O,-0.2986817119,-1.2648745317,1.2580338099
O,-0.2986817119,-1.2648745317,-1.2580338099
C,0.0476052262,2.1702225442,0.
O,-0.3333657988,3.2426927266,0.

so2_h2cch2_2 MP2= -626.37432410 NIMAG= 0
S,0.4618124817,-1.0285174489,-0.0507989272
O,0.0185288455,-1.2261192066,1.3301509993
O,-0.5239015573,-1.1662950225,-1.1240767111
C,-0.1088612777,2.2942404947,-0.6917555007
C,-0.3604429721,2.1632695718,0.6125688692
H,-0.7750641783,1.8806488966,-1.4364528384
H,-1.239346378,1.6414714607,0.9662671767
H,0.7686073911,2.8186146659,-1.0446004782
H,0.3046446447,2.5758285882,1.358697408

so2_h2cch2 MP2= -626.37430980 NIMAG= 1
S
X,1,1.
O,1,r1,2,a1
O,1,r1,2,a1,3,180.,0
X,1,r2,2,a2,3,90.,0
C,5,r3,1,a3,2,90.,0
C,5,r3,1,a3,2,-90.,0
H,6,r4,5,a4,1,d4,0
H,7,r4,5,a4,1,-d4,0
H,6,r5,5,a5,1,d5,0
H,7,r5,5,a5,1,-d5,0

r1=1.46371314

a1=120.81556935
r2=3.22720468
a2=88.45226798
r3=0.67627998
a3=99.30373133
r4=1.08140609
a4=118.32748467
d4=68.47660464
r5=1.08140579
a5=123.51890416
d5=-100.73420218

so2_hcch MP2= -625.13375396 NIMAG= 0
S
X,1,1.
O,1,r1,2,a1
O,1,r1,2,a1,3,180.,0
X,1,r2,2,a2,3,90.,0
C,5,r3,1,a3,2,90.,0
C,5,r3,1,a3,2,-90.,0
H,5,r4,1,a4,2,90.,0
H,5,r4,1,a4,2,-90.,0

r1=1.46347905
a1=120.75364227
r2=3.3637298
a2=90.27086261
r3=0.61401663
a3=81.07215814
r4=1.67215524
a4=86.59106537

so2_n2 MP2= -657.33218722 NIMAG= 0
S
X,1,1.
O,1,r1,2,a1
O,1,r1,2,a1,3,180.,0
N,1,r2,2,a2,3,90.,0
N,1,r3,2,a3,3,90.,0

r1=1.46358594
a1=120.68685587
r2=3.2895353
a2=102.82989998
r3=4.33835361
a3=108.53343525

so2_nch MP2= -641.23069894 NIMAG= 0
S,0.4076065263,-1.0493616808,0.
O,-0.3346385345,-1.1675376943,1.2559172514
O,-0.3346385345,-1.1675376943,-1.2559172514

N,0.2225078271,1.9575052173,0.
C,-0.3277866868,2.9856844421,0.
H,-0.8324310341,3.923872813,0.

so2_nh3 MP2= -604.43470368 NIMAG= 0
S
X,1,1.
O,1,r1,2,a1
O,1,r1,2,a1,3,180.,0
N,1,r2,2,a2,3,90.,0
H,5,r3,1,a3,3,d3,0
H,5,r3,1,a3,4,-d3,0
H,5,r4,1,a4,2,0.,0

r1=1.4642463
a1=121.22123334
r2=2.76340632
a2=85.72390264
r3=1.01357169
a3=100.9875292
d3=-3.7671016
r4=1.01279738
a4=131.1970961

so2_oh2 MP2= -624.30091677 NIMAG= 0
S
X,1,1.
O,1,r1,2,a1
O,1,r1,2,a1,3,180.,0
O,1,r2,2,a2,3,90.,0
H,5,r3,1,a3,3,d3,0
H,5,r3,1,a3,4,-d3,0

r1=1.46346042
a1=120.95998473
r2=2.84976501
a2=88.19482725
r3=0.96294437
a3=106.12286975
d3=-3.70282716

so2_ph3 MP2= -890.63030785 NIMAG= 0
S
X,1,1.
O,1,r1,2,a1
O,1,r1,2,a1,3,180.,0
P,1,r2,2,a2,3,90.,0
H,5,r3,1,a3,2,d3,0
H,5,r3,1,a3,2,-d3,0
H,5,r4,1,a4,2,180.,0

r1=1.46390255
a1=120.83105719
r2=3.51249237
a2=92.14804799
r3=1.4110183
a3=132.30160675
d3=83.17961589
r4=1.41137858
a4=90.79078934

so2_sh2 MP2= -946.87896952 NIMAG= 0
S
X,1,1.
O,1,r1,2,a1
O,1,r1,2,a1,3,180.,0
S,1,r2,2,a2,3,90.,0
H,5,r3,1,a3,3,d3,0
H,5,r3,1,a3,4,-d3,0

r1=1.46424883
a1=120.9046831
r2=3.41449069
a2=91.38117819
r3=1.33773451
a3=76.8050595
d3=-11.35010619

YB complexes with SeO_2 as LA.

seo2_c3h6 MP2= -2667.95693699 NIMAG= 0
Se,0.4382213818,-1.4082146958,0.
O,-0.4212786551,-1.6160141568,1.3595720326
O,-0.4212786551,-1.6160141568,-1.3595720326
C,0.7430950913,1.703313116,0.
C,-0.7562805631,1.494164745,0.
H,1.2824937473,1.5004133821,-0.9137535695
H,-1.2061222881,1.1314640114,-0.9123190517
H,1.2824937473,1.5004133821,0.9137535695
H,-1.2061222881,1.1314640114,0.9123190517
C,-0.1929831429,2.8843879648,0.
H,-0.2688410624,3.4556911184,-0.9120746979
H,-0.2688410624,3.4556911184,0.9120746979

seo2_ch2o MP2= -2664.65180948 NIMAG= 0
Se,-0.953559279,-0.038941105,0.4289287292
O,-1.2699114564,1.4201233146,-0.1974294434
O,-0.69909811,-1.1753370984,-0.702256418
O,1.6933727743,0.2863811446,0.5727873928
C,2.1371235502,-0.2161861568,-0.444787912
H,1.4807963047,-0.7603664456,-1.1387183803

H,3.2029922161,-0.1348276532,-0.6869459682

seo2_co MP2= -2663.46848761 NIMAG= 0
Se,0.5228241213,-1.1334834439,0.
O,-0.3493658969,-1.2645329371,1.3614436362
O,-0.3493658969,-1.2645329371,-1.3614436362
C,0.1015532989,2.1730071882,0.
O,-0.3686648166,3.2092636052,0.

seo2_h2cch2 MP2= -2628.73334605 NIMAG= 0
Se,0.3557839013,-0.7137689734,-0.0784706802
O,-0.0048938558,-1.0135226042,1.4738768937
O,-0.8999048227,-0.8857917748,-1.0893665595
C,-0.2513446575,2.5604291823,-0.7074970923
C,-0.5751447438,2.3737395988,0.5744555252
H,-0.8009699476,2.0703918215,-1.5000406047
H,-1.3980583045,1.7309232027,0.8578189603
H,0.5670652654,3.2076859326,-0.9919698304
H,-0.0297018347,2.8605996147,1.3711933881

seo2_hcch MP2= -2627.49231018 NIMAG= 0
Se
X,1,1.
O,1,r1,2,a1
O,1,r1,2,a1,3,180.,0
X,1,r2,2,a2,3,90.,0
C,5,r3,1,a3,2,90.,0
C,5,r3,1,a3,2,-90.,0
H,5,r4,1,a4,2,90.,0
H,5,r4,1,a4,2,-90.,0

r1=1.62104038
a1=122.91826249
r2=3.24855964
a2=85.66186014
r3=0.60672546
a3=88.99436962
r4=1.66970954
a4=89.46040544

seo2_n2 MP2= -2659.69011633 NIMAG= 0
Se
X,1,1.
O,1,r1,2,a1
O,1,r1,2,a1,3,180.,0
N,1,r2,2,a2,3,90.,0
N,1,r3,2,a3,3,90.,0

r1=1.62253174

a1=122.89111442
r2=3.27951439
a2=100.38297002
r3=4.31338022
a3=106.71326436

seo2_nch MP2= -2643.59006914 NIMAG= 0
Se,0.4772229869,-0.9919621692,0.
O,-0.3892629065,-1.1776329306,1.3574373182
O,-0.3892629065,-1.1776329306,-1.3574373182
N,0.2631099515,1.9684223735,0.
C,-0.3186327124,2.9787599691,0.
H,-0.8534323517,3.9002578313,0.

seo2_nh3 MP2= -2606.79669656 NIMAG= 0
Se
X,1,1.
O,1,r1,2,a1
O,1,r1,2,a1,3,180.,0
N,1,r2,2,a2,3,90.,0
H,5,r3,1,a3,3,d3,0
H,5,r3,1,a3,4,-d3,0
H,5,r4,1,a4,2,0.,0

r1=1.6190877
a1=123.41077116
r2=2.61652189
a2=86.82386292
r3=1.01442269
a3=99.85423891
d3=-1.51725287
r4=1.01308331
a4=131.06951299

seo2_oh2 MP2= -2626.66095437 NIMAG= 0
Se,-0.3872485228,0.3540436146,0.
O,0.4587623716,0.6162428367,1.3560825252
O,0.4587623716,0.6162428367,-1.3560825252
O,0.4141007013,-2.2913595867,0.
H,1.0045615391,-2.3045483506,0.7622404215
H,1.0045615391,-2.3045483506,-0.7622404215

seo2_ph3 MP2= -2892.98939002 NIMAG= 0
Se,-0.4445252973,0.7814707338,0.0063242139
O,0.4342020614,1.2960589483,1.2686935762
O,0.4146626538,0.5970428226,-1.3581960682
P,0.3893276122,-2.45743707,0.1418390158
H,0.5285448617,-2.6886779649,-1.2422145839
H,0.6535807876,-3.7889062713,0.5261869116

H,1.7139643206,-2.0048091984,0.3173669345

seo2_sh2 MP2= -2949.23846982 NIMAG= 0
Se
X,1,1.
O,1,r1,2,a1
O,1,r1,2,a1,3,180.,0
S,1,r2,2,a2,3,90.,0
H,5,r3,1,a3,3,d3,0
H,5,r3,1,a3,4,-d3,0

r1=1.62177408
a1=123.16822422
r2=3.31045614
a2=90.15539151
r3=1.3386648
a3=75.29403453
d3=-8.6910856

Table S6. Linear correlations of D_e vs. the interatomic distance (R^2 coefficients)

Hydrogen bonded complexes

Correlation for all complexes of a given Lewis base (n= 6)		Correlation for all complexes of a given Lewis acid (n= 11)	
Lewis Base	R^2	Lewis Acid	R^2
N_2	0.94	HF	0.62
C≡O	0.91	HBr	0.80
HC≡CH	0.90	HCl	0.73
$H_2C=CH_2$	0.90	HC≡N	0.61
C_3H_6	0.53	H2O	0.68
PH_3	0.87	HC≡CH	0.53
H_2S	0.89		
HC≡N	0.83		
$H_2C=O$	0.86		
H_2O	0.85		
NH_3	0.76		

Halogen bonded complexes

Correlation for all complexes of a given Lewis base (n= 5)		Correlation for all complexes of a given Lewis acid (n= 11)	
Lewis Base	R^2	Lewis Acid	R^2
N_2	0.11	ClF	0.82
C≡O	0.17	ClBr	0.67
HC≡CH	0.02	Br_2	0.71
$H_2C=CH_2$	0.18	Cl_2	0.64
C_3H_6	0.32	F_2	0.53
PH_3	0.95		
H_2S	0.40		
HC≡N	0.14		
$H_2C=O$	0.13		
H_2O	0.08		
NH_3	0.68		

Tetrel bonded complexes

Correlation for all complexes of a given Lewis base (n= 4)		Correlation for all complexes of a given Lewis acid (n= 11)	
Lewis Base	R^2	Lewis Acid	R^2
N_2	0.03	GeH_3F	0.62
C≡O	0.10	SiH_3F	0.70
HC≡CH	0.05	$F_2C=O$	0.62
$H_2C=CH_2$	0.09	CO_2	0.58
C_3H_6	0.02		
PH_3	0.22		
H_2S	0.02		
HC≡N	0.25		

$H_2C{=}O$	0.47		
H_2O	0.00		
NH_3	0.61		

Pnictogen bonded complexes

Correlation for all complexes of a given Lewis base (n= 4)		Correlation for all complexes of a given Lewis acid (n= 11)	
Lewis Base	R^2	Lewis Acid	R^2
N_2	0.28	AsH_2F	0.29
C≡O	0.90	PH_2F	0.39
HC≡CH	0.07	NO_2F	0.47
$H_2C{=}CH_2$	0.85	N_2O	0.46
C_3H_6	0.02		
PH_3	0.88		
H_2S	0.96		
HC≡N	0.86		
$H_2C{=}O$	0.84		
H_2O	0.61		
NH_3	0.87		

Chalcogen bonded complexes

Correlation for all complexes of a given Lewis base (n= 4)		Correlation for all complexes of a given Lewis acid (n= 11)	
Lewis Base	R^2	Lewis Acid	R^2
N_2	0.29	SeF_2	0.68
C≡O	0.62	SeO_2	0.84
HC≡CH	0.57	SF_2	0.74
$H_2C{=}CH_2$	0.59	SO_2	0.63
C_3H_6	0.30		
PH_3	0.92		
H_2S	0.76		
HC≡N	0.63		
$H_2C{=}O$	0.59		
H_2O	0.69		
NH_3	0.86		

Table S7. $V_{S,min}$ and V_{min} (a.u.) of the Lewis Bases and $V_{S,max}$ (a.u.) of the Lewis acids. The 0.001 au electron density isosurface has been used to calculate $V_{S,min}$ and $V_{S,max}$.

Lewis base	$V_{S,min}$	V_{min}
N_2	-0.0136	-0.0155
CO	-0.0223	-0.0278
HC≡CH	-0.0233	-0.0283
$H_2C=CH_2$	-0.0235	-0.0294
C_3H_6	-0.0193	-0.0237
H_3P	-0.0256	-0.0300
H_2S	-0.0264	-0.0317
HN≡C	-0.0509	-0.0661
$H_2C=O$	-0.0462	-0.0610
H_2O	-0.0515	-0.0708
H_3N	-0.0594	-0.0979

Lewis acids, $V_{S,max}$

HF	0.1096	ClF	0.0654	SO_3	0.0840	AsH_2F	0.0684	GeH_3F	0.0677
HBr	0.0610	BrCl	0.0538	SeF_2	0.0735	PH_2F	0.0603	SiH_3F	0.0619
HCl	0.0724	Br_2	0.0452	SeO_2	0.0544	NO_2F	0.0587	$F_2C=O$	0.0675
HC≡N	0.0826	Cl_2	0.0407	SF_2	0.0598	N_2O	0.0347	CO_2	0.0414
H_2O	0.0709	F_2	0.0264	SO_2	0.0496				
HC≡CH	0.0513								

Table S8. Linear correlations of D_e vs. the MEP parameters ($V_{S,max}$, $V_{S,min}$ and V_{min}) (R^2 coefficients)

Correlation for all complexes of a given Lewis base vs. $V_{S,max}$ of the Lewis acid (n= 24)

Lewis Base	R^2
N_2	0.40
C≡O	0.51
HC≡CH	0.38
$H_2C{=}CH_2$	0.30
C_3H_6	0.27
PH_3	0.25
H_2S	0.38
HC≡N	0.75
$H_2C{=}O$	0.53
H_2O	0.69
NH_3	0.43

Correlation for all complexes of a given Lewis acid (n= 11) vs. the $V_{S,min}$ and V_{min} of the Lewis bases

Lewis Acid	R^2 (V_{min})	R^2 ($V_{S,min}$)	Lewis acid	R^2 (V_{min})	R^2 ($V_{S,min}$)
HF	0.91	0.97	SO_3	0.57	0.69
HBr	0.76	0.84	SeF_2	0.70	0.77
HCl	0.85	0.92	SeO_2	0.70	0.74
HC≡N	0.95	0.96	SF_2	0.78	0.83
H_2O	0.89	0.91	SO_2	0.74	0.75
HC≡CH	0.88	0.89			
			AsH_2F	0.60	0.64
ClF	0.37	0.43	PH_2F	0.64	0.67
BrCl	0.58	0.68	NO_2F	0.73	0.74
Br_2	0.54	0.63	N_2O	0.56	0.57
Cl_2	0.66	0.74			
F_2	0.68	0.74	GeH_3F	0.82	0.88
			SiH_3F	0.82	0.90
			$F_2C{=}O$	0.82	0.82
			CO_2	0.68	0.67
